# Human brain tissue with MOGHE carrying somatic *SLC35A2* variants reveal aberrant protein expression and protein loss in the white matter

**DOI:** 10.1007/s00401-025-02858-7

**Published:** 2025-03-05

**Authors:** Erica Cecchini, Simon Geffers, Roland Coras, Dorothea Schultheis, Christian Holtzhausen, Kristina Karandasheva, Harald Herrmann, Friedrich Paulsen, Christine Stadelmann, Katja Kobow, Till Hartlieb, Christian G. Bien, Dennis Lal, Ingmar Blumcke, Lucas Hoffmann

**Affiliations:** 1 Department of Neuropathology, Partner of the European Reference Network (ERN) EpiCARE, Universitätsklinikum Erlangen, Friedrich-Alexander Universität (FAU) Erlangen-Nürnberg, Erlangen, Germany; 2 Institute of Functional and Clinical Anatomy, FAU Erlangen-Nürnberg, Erlangen, Germany; 3 Department of Neuropathology, University Medical Center Göttingen, Göttingen, Germany; 4 Center for Pediatric Neurology, Neurorehabilitation, and Epileptology, Schoen-Clinic, Vogtareuth, Germany; 5 Research Institute for Rehabilitation, Transition, and Palliation, Paracelsus Medical University, Salzburg, Austria; 6 Department of Epileptology, Krankenhaus Mara, Bethel Epilepsy Center, Medical School OWL, Bielefeld University, Bielefeld, Germany; 7 Department of Neurology, The University of Texas Health Science Center at Houston, Houston, TX USA; 8 Center for Neurogenetics, The University of Texas Health Science Center at Houston, Houston, TX USA

**Keywords:** MOGHE, Neuropathology, Brain, Myelin, Oligodendrocytes, Epilepsy

## Abstract

Mild Malformation of Cortical Development with Oligodendroglial Hyperplasia in Epilepsy (MOGHE) is a recently described disease entity primarily affecting young children with drug-resistant epilepsy, mainly affecting the frontal lobe. The condition is histopathologically defined by focal lesions with patchy areas of increased oligodendroglial cell density at the grey-white matter boundary and heterotopic neurons in the white matter. Approximately half of the individuals with MOGHE carry brain somatic variants in the *SLC35A2* gene, which affects the UDP-galactose transporter and thus sphingolipid glycosylation. To investigate the impact of *SLC35A2* variants on protein expression, we analysed MOGHE brain tissue with and without *SLC35A2* mosaicism, distinguishing missense from nonsense variants. We developed an antibody targeting the N-terminus of the SLC35A2 galactose transporter and applied it for immunofluorescence (IF) analyses in a MOGHE cohort comprising 59 genetically tested individuals selected from three centres in Germany. The cohort included 13 individuals with *SLC35A2* missense variants and 15 with *SLC35A2* nonsense variants. Our findings confirm the localisation of the SLC35A2 protein in the Golgi apparatus of all neuroepithelial cell types as well as within Golgi outposts along oligodendroglial processes. The protein distribution was altered in MOGHE samples dependent on the *SLC35A2* variant and its allelic frequency. Western blot and IF analyses revealed a significant SLC35A2 reduction in MOGHE tissues carrying nonsense variants. Ultrastructural analyses from three MOGHE samples demonstrated hypomyelination in regions with increased oligodendroglial cell densities, regardless of the harbouring of *SLC35A2* variants. Notably, this hypomyelination pattern decreased with age. These results suggested a role for the SLC35A2 protein in the pathogenesis of MOGHE and indicated the presence of additional myelin-associated pathomechanisms in those individuals who do not carry a pathogenic *SLC35A2* variant.

**Supplementary Information:**

The online version contains supplementary material available at 10.1007/s00401-025-02858-7.

## Introduction

Mild Malformation of Cortical Development with Oligodendroglial Hyperplasia in Epilepsy (MOGHE) is a novel disease entity of drug-resistant epilepsy mainly in the pediatric population, with a predominant localization in the frontal lobe [36]. Its histopathology is characterized by clusters of increased oligodendroglial cell densities at the grey-white matter junction [7, 17, 19, 43] and heterotopic neurons in the white matter. Patients present with two different phenotypes: (1) early-onset epileptic encephalopathy (EE) characterized by epileptic spasms and significant cognitive impairment and (2) drug-resistant focal epilepsy (DR-FE) with onset in adolescence or young adulthood and normal/borderline cognitive levels [3]. The 2022 classification update of focal cortical dysplasia (FCD) by the International League against Epilepsy (ILAE) defines MOGHE as a white matter abnormality [30]. In early and later childhood it presents neuroradiologically with two different, age-dependent subtypes: (1) with juxta-cortical laminar hyperintensities up to the age of 5 years and (2) with reduced cortico-medullary differentiation in older individuals [20]. In adult patients aberrant cortical folding, cortical thickening and cleft cortical dimples were observed[29].

Recent studies used deep-targeted gene sequencing of cerebral MOGHE tissue samples obtained from multicentric cohorts of pediatric individuals to identify the molecular pathogenesis of the condition [3, 6, 7, 27]. These analyses provided ample evidence for brain somatic pathogenic variants within *SLC35A2* in almost half of the individuals examined, with variant allele frequencies ranging from 2 to 52% [7]. The *SLC35A2* gene is located on the X chromosome (Xp11.23) and encodes a UDP-galactose transporter central in moving galactose from the cytosolic compartment into the lumen of the Golgi apparatus. Transportation of galactose into the Golgi apparatus is crucial for post-translational modification, e.g., glycosylation of proteins and lipids. This may apply particularly to sphingolipids as the primary contributors to myelin formation and stability [34]. Hence alterations in sphingolipid glycosylation can profoundly impact the adhesion of the lipid bilayer around the axon.

Supporting the hypothesis of a pathogenic role of *SLC35A2* in MOGHE, brain somatic mutations in *SLC35A2* cause intractable epilepsy with aberrant *N*-glycosylation in affected brain tissue [37], whereas germline variants give rise to congenital disorders of glycosylation, i.e., SLC35A2-CDG. This multiorgan syndrome includes neurodevelopmental features, some of which resemble those described in MOGHE, e.g., epileptiform activity and structural brain abnormalities on MRI [29, 42]. In addition, a recent *SLC35A2* knockout in the *Olig2-* lineage of a mouse model demonstrated a direct causal role for *SLC35A2* in MOGHE-like phenotypes and recapitulated the increased oligodendroglial cell density [47].

The *SLC35A2* gene represents a novel molecular pathway in MCDs that is distinct from the previously described gene variants, which have so far focused on the MTOR pathway in focal cortical dysplasia (FCD). [5, 10, 27]. The diagnosis of MOGHE is increasingly recognized in cohorts of individuals with drug-resistant focal epilepsy [2, 3, 12, 18, 26, 46], it is therefore, of utmost importance to better understand the molecular pathogenesis of *SLC35A2-altered* MOGHE, as it represents a potential target for personalized treatment, e.g., supplementation of D-galactose [1]. D-galactose supplementation has already been established in young children diagnosed with SLC35A2-CDG [45]. Our study better characterizes the distribution of the SLC35A2 protein in MOGHE, evaluates the impact of variant types on protein expression levels, and investigates whether similar histopathological patterns are present in MOGHE cases with or without genetically confirmed *SLC35A2* variants.

## Methods

### Case cohort

We retrieved a cohort of 59 individuals from the archive of the Neuropathological Institute of the University Hospital Erlangen selected from three centres in Germany and previously reported in [3, 25, 27] and [8]. The cohort comprised 38 males (64%) and 21 females (36%; Table 1) with 15 individuals harbouring *SLC35A2* nonsense variants (MOGHE^*SLC35A2*nonsense^) and 13 individuals harbouring missense variants (MOGHE^*SLC35A2*missense^). The variant allele frequencies (VAF) ranged from 3 to 52%, as reported in [25] (Supplementary Table 1A). No *SLC35A2* variants (noVar.) were identified in the remaining 31 individuals (MOGHE^*SLC35A2*noVar.^). Formalin-fixed and paraffin-embedded (FFPE) tissue blocks were microscopically examined by two expert neuropathologists (Ingmar Bluemcke and Roland Coras) following the ILAE work-up guidelines for epilepsy surgery specimens [4] and the latest ILAE consensus classification of Focal Cortical Dysplasia [30]. We selected 30 age-matched brain specimens as controls, including 10 with frontal lobe resections for epilepsy due to Focal Cortical Dysplasia ILAE type IIa (FCDIIa), 10 with temporal lobe resections due to temporal lobe epilepsy (TLE) and 10 samples of the frontal neocortex obtained from age-matched post-mortem specimens of individuals with no reported seizure disorder (Supplementary Table 1B). Eight out of ten samples from our FCDIIa cohort were submitted for gene panel sequencing. No pathogenic variants in *SLC35A2* were detected in seven samples, while one sample was excluded due to insufficient DNA quality. The three FCDIIa cases without *SLC35A2* genetic data were used exclusively for oligodendroglial cell quantification and were not included in the Western blot or immunofluorescence analyses. Two samples from individuals with temporal lobe epilepsy (TLE) underwent FCD gene panel sequencing, revealing no *SLC35A2* variants (Supplementary Table 1B). None of the individuals with TLE had a clinical history of epileptic encephalopathy. To date, *SLC35A2* variants have not been reported in TLE or TLE with hippocampal sclerosis (TLE-HS) [8, 9, 27]. Therefore, samples from individuals with TLE-HS were deemed appropriate and reliable control samples for this study. We extracted the following clinical variables from the database: mutational status of the *SLC35A2* and relative variant allele frequency (VAF), brain region (lobe), age at epilepsy onset, age at surgery, and postsurgical seizure outcome according to Engel's classification. The study was approved by the ethical review boards of the Medical Faculty of the Friedrich-Alexander University of Erlangen-Nuremberg (#193_18B, 18-192_1-Bio) and the University of Münster (2015-088-f-S). The cohort data were used to calculate statistical analyses on the lesion-associated brain region, outcome after surgery, age at onset, age at surgery and the positive or negative harbouring of a variant within *SLC35A2*. Twenty MOGHE samples with and without *SLC35A2* variants were selected for statistical analysis of oligodendroglial cell densities and to create heat maps**.** Four MOGHE samples with nonsense *SLC35A2* variants were selected for Western blotting. Forty-two samples from the MOGHE cohort were used for IF analyses and representative images were selected to build the Figure panels. Three samples from MOGHE individuals aged 5, 7 and 44 have been used for the acquisition of electron microscopy images and myelin content quantifications. Two of these samples carried an *SLC35A2* missense variant.

**Table 1:** Individuals included in the study

Cohort	Males (*n*)	Females (*n*)	Age at first surgery (years)	Age of epilepsy onset (years)
MOGHE				
*SLC35A2* noVar	20	11	11 (3–35)	2.3 (0–12)
*SLC35A2* missense	7	6	15.8 (2–59)	4.9 (0–24)
*SLC35A2* nonsense	11	4	8.1 (4–43)	2.3 (0–12)
Control				
TLE	8	2	21.5 (5–36)	15.2 (4–28)
FCDIIa	5	5	10.3 (3–19)	2.3 (0–4)
Autopsy	8	2	16.9 (0–49)*	n.a

Individuals included in the study. noVar.: tissue samples without *SLC35A2* variants as reported in [8, 25, 27]; Missense: tissue samples positive for *SLC35A2* missense variants as reported in [3, 25, 27]; Nonsense: tissue samples positive for *SLC35A2* nonsense variants as reported in [3, 25, 27]; Age at first surgery, age of epilepsy onset, and duration of epilepsy are reported as the group average, followed by the age range in brackets

*TLE* temporal lobe epilepsy. *FCDIIa* focal cortical dysplasia ILAE type IIa

*For autopsy controls, age at first surgery refers to the age of death

### Antibodies directed against SLC35A2 and immunofluorescence analyses

We designed a polyclonal antibody directed against the first 28 N-terminal amino acids of the SLC35A2 transporter (AAVGAGGSTAAPGPGAVSAGALEPGTAS) coupled to hemocyanin via an N-terminal cysteine to study the localization of SLC35A2 protein in human brain samples by IF methods. The antibody was produced by Peptide Specialty Laboratories GmbH (Germany) by immunizing two rabbits (Rb1 and Rb2) with the above antigen complex, and each serum was affinity purified by column chromatography with the above peptide coupled to Sepharose. The specificity of the two antibodies was separately tested by Western blotting at a final dilution of 1:100 and verified by comparison with His-Taq labelled antibodies against the *E. coli* induced protein of SLC35A2. A commercial SLC35A2 antibody from Abcam company (ab222854) was tested and compared to the newly generated one to validate the staining pattern (Supplementary Fig. 1).

Double IF analyses of SLC35A2 coupled to major cell markers were performed using antibodies directed against the anti-2′,3′-Cyclic-nucleotide 3'-phosphodiesterase (CNPase), anti-Oligodendrocyte transcription factor 2 (Olig2), anti-58k Golgi protein, anti-Tubulin Polymerization-Promoting Protein (TPPP), a marker for Golgi outpost, anti-Neuronal Nuclei (NeuN), anti-Microtubule-Associated Protein 2 (MAP2), anti-Novel Amplified in Breast Cancer 1 (NABC1 or BCAS1), and anti-glial fibrillary acidic protein (GFAP). All fluorescence-labeled secondary antibodies were used according to the manufacturer's protocols (Supplementary Table 2).

### Confocal microscopy and 3D reconstructions

High-resolution z-stacks were taken utilizing the Leica Stellaris 8 confocal microscope in lightning settings with further deconvolution. Z-stack post-processing was performed using Fiji by ImageJ (Version 2.14.0/1.54f). 3D reconstruction of entire z-stacks was performed utilizing the open-source software 3D Slicer (Version 5.7.0) and the Volume Rendering PlugIn.

### Immunoblotting

Frozen brain specimens from four MOGHE individuals with *SLC35A2* nonsense variants were sectioned at 20 µm thickness and then suspended in lysis buffer (5% SDS; 15% glycerol; 50 mM Na_3_PO_4;_ 5mM EDTA; 5mM EGTA; 40 mM DTT; 3 mM Pefabloc, Sigma Aldrich #76307; 0.75 mM PMSF; 1 × Complete, Sigma Aldrich #11873580001; 1:300 Benzonase Sigma-Aldrich, #E1014-25KU). Tissue microdissection was performed from the white matter in MOGHE and controls. In MOGHE samples, the region of interest was microscopically defined by clusters of increased oligodendroglial cell density. Suspended samples were heated to 95 °C and then centrifuged at 13,000 g for 15 min. Supernatants were collected and the remaining pellets were discarded. Immediately before electrophoresis, samples were diluted in Laemmli buffer [24]. The samples were subjected to a routine SDS-PAGE protocol using self-cast gels (12.5% polyacrylamide). Protein transfer was done using wet tank transfer (Towbin buffer) onto PVDF membranes. Immediately after the transfer, membranes were rinsed in deionized water, and then air dried overnight. Before detection, dried PVDF membranes were stained using the Sypro Ruby total protein stain (Invitrogen, S11791) according to manufacturer instructions. Staining was visualized under UV light. For chemiluminescent detection, membranes were submerged in methanol, rinsed briefly, and then blocked in 5% milk powder suspended in TBS-T for 60 min on a horizontal shaker. Primary antibodies were diluted in 5% milk powder + TBS-T, membranes were incubated for 60 min at RT and then washed in TBS-T for 3 × 10 min. The secondary antibody was diluted in TBS-T and incubated for 30 min at RT, followed by another washing step. Three ml of ECL solution (West Pico ECL, ThermoScientific) were applied to each membrane and incubated for 5 min. Images were captured using the Chemostar Touch CCD system (Intas GmbH, Göttingen, Germany). Densitometric analysis was performed using ImageLab 1D (L340) and Fiji (by ImageJ, Version 2.14.0/1.54f). All experiments were performed as independent triplicates. Alpha-tubulin (Sigma-Aldrich, T5168) was used as the loading control, and the mean of triplicate values was used for statistical analysis.

### Digital microscopy analyses

The SLC35A2 signal quantification represented in the graph in Fig. 3 was conducted using QuPath v0.5.1 across four genetically defined groups: TLE, MOGHE^*SLC35A2*missense^, MOGHE^*SLC35A2*nonsense^, and MOGHE^*SLC35A2*noVar^, with four independent samples per group, and statistically analyszed using the Multiple comparison Welch’s ANOVA test. A thresholder was created to annotate the signal by setting a threshold value that distinguished pixels based on intensity. Pixels with values above the threshold were classified as positive, representing the SLC35A2 signal. The thresholding was performed manually and consistetntly applied across all images and the threshold value was carefully chosen to exclude any background. All detected pixels were transformed into annotations to extract measurement data. For each sample, the area of the SLC35A2 signal was measured within predefined regions of interest (ROIs) of consitant size (880 µm^2^) within areas of high oligodendroglial cell density. Three ROIs were analyzed per each case, and the mean signal area of the three measurements was used for the generation of the graph. To generate heatmaps of oligodendroglial cell densities,, oligodendrocytes from FFPE tissue sections were immunohistochemically labelled with anti-Olig2 antibodies. Slides were then digitally scanned with a Hamamatsu Nanozoomer S60 (Japan) and visualized with NDP view 2.0 digital microscopy software. Oligodendrocytes were automatically detected using a calculation pipeline with QuPath v0.4.4 software for imaging analysis. The heat map colour scale (Jet Legacy) indicated maximum oligodendrocyte density in red and the lowest density in green. Statistical analyses and diagram creation were performed using GraphPad Prism 8. The analyses of myelin density were assessed semi-quantitatively using Nissl-LFB staining. MOGHE lesions were compared with non-lesional areas within the same sample to account for intrinsic staining variables, such as patient age, chemical incubation times, and sample sensitivity. The colour intensity of laminae I-II of the neocortex served as a reference. Five ROIs within the white matter were selected from each slide and each group to assess relative intensity differences. These differences were then correlated with patient age and compared across samples. For MOGHE samples, all ROIs were sampled from lesion areas defined by clustered areas of increased oligodendroglial cell density. Anti-NABC1 antibodies detecting Brain Enriched Myelin Associated Protein 1 (BCAS1, formerly known as Breast carcinoma-amplified sequence 1) were used to assess the presence of premyelinating and actively myelinating oligodendrocytes. Fluorescence labelling was recorded with the Hamamatsu Nanozoomer S60 equipped with a fluorescence lamp (Hamamatsu LX2000) and post-scanning image processing was conducted using QuPath 0.4.4.

### Electron microscopy

Surgical brain tissue from three MOGHE individuals was available for further analysis by electron microscopy. This included two females with MOGHE carrying *SLC35A2* variants (ID 33 and 43) and one male with MOGHE^*SLC35A2*noVar.^ (ID 7). We applied transmission electron microscopy (TEM) using an intra-sample evaluation, comparing lesion areas from MOGHE samples with perilesional areas as a reference for control conditions. The three MOGHE samples were fixed in Ito’s fixative consisting of 2.5% glutaraldehyde, 2.5% paraformaldehyde, and 0.2% picric acid dissolved in cacodylate buffer, pH 7.3 for 1 week following embedding in Epon. Semi-thin Sects. (1 μm) were cut with an ultramicrotome (Ultracut Leica, Wetzlar, Germany) and stained with toluidine blue. Ultrathin sections were stained with uranyl acetate and lead citrate and viewed with a transmission electron microscope Zeiss TEM 902 ESI (Carl Zeiss, Jena, Germany). Further g-ratio calculations were performed using the manual QuPath v.0.5.2 line annotation tool and GraphPad Prism 8 for statistical analysis on 100 axons per lesional/perilesional group from four different samples (IDs 7, 33, 34, and 47). Only completely visible, transversely cut axons were annotated. g-Ratio calculations were based on the whole diameter of the axons compared to the whole diameter of the axons plus the myelin sheet.

## Results

Fifty-nine patients were histopathologically diagnosed with MOGHE using the ILAE classification scheme [30], had affected tissue submitted to genotyping (see [1, 3, 7, 27] for further details), and were included in the study. In 68% of cases, the lesion was localized to the frontal lobe, while the temporal lobe was affected in 12% of cases. In the remaining individuals, pre-surgical evaluations revealed a more extensive epileptic region, involving two or more lobes. Our three genetically defined MOGHE groups did not differ significantly for age at disease onset or age at surgery (Table 1). However, it is noteworthy that individuals carrying *SLC35A2* nonsense variants experienced an earlier onset of epilepsy at an average age of 2.3 years, compared to 4.9 years for those with missense variants. Follow-up data, including postsurgical outcomes according to Engel’s classification, were available for 46 patients (Supplementary Table 1A). Sixty-one per cent of individuals with MOGHE^*SLC35A2*noVar.^. had an Engel Outcome class I compared to the 66% in individuals with MOGHE^*SLC35A2*nonsense^ and the 60% in individuals with MOGHE^*SLC35A2*missense^.

### 1. SLC35A2 protein is located within the Golgi apparatus and Golgi outposts along oligodendroglial processes

**Fig. 1:**
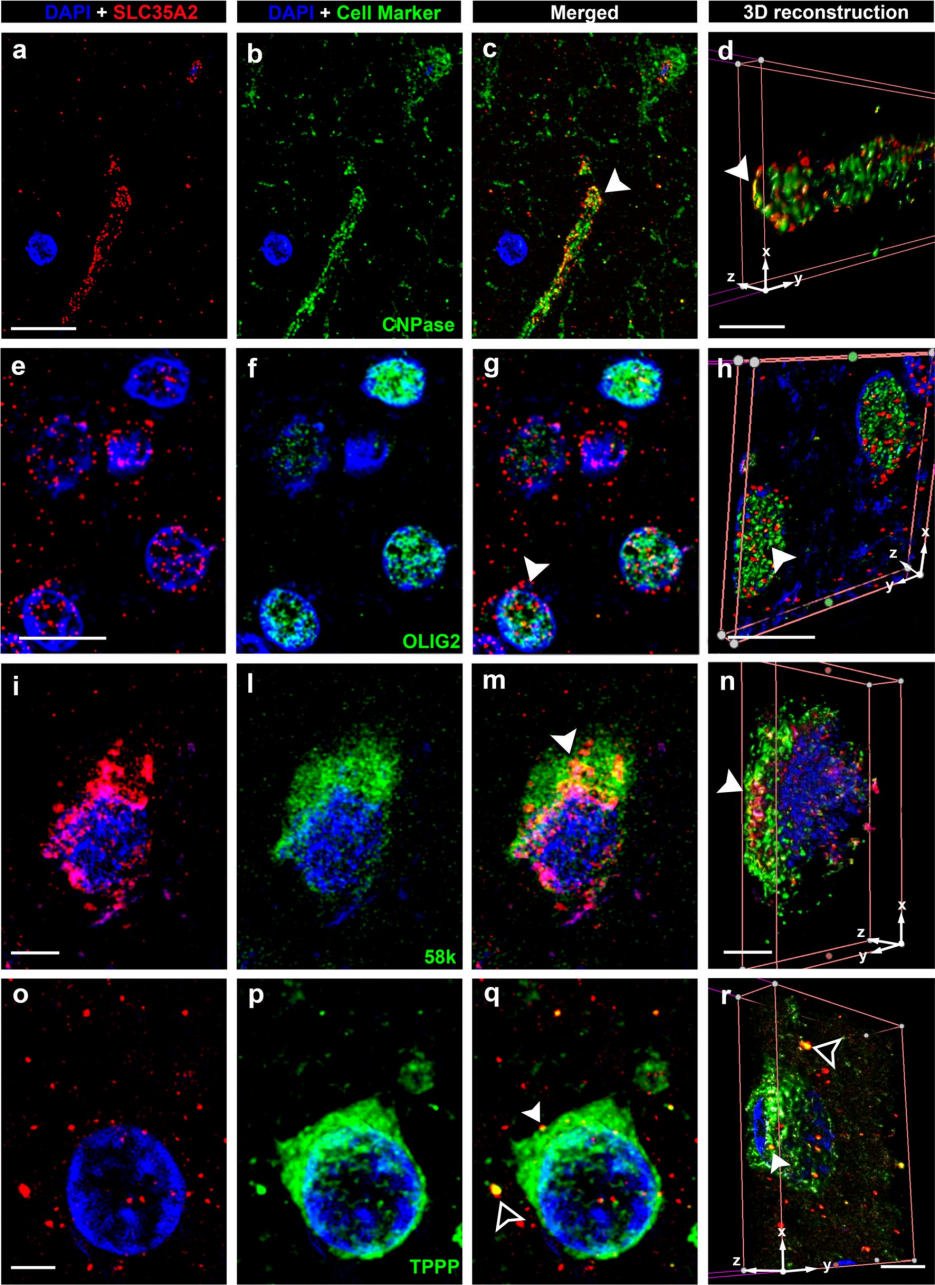
Confocal laser scanning microscopy of SLC35A2. Representative immunofluorescent images arranged in a 4 × 4-panel format. Panels **a, e, i,** and** o** on the far left display DAPI (blue nuclear stain) alongside SLC35A2 immunoreactivity (in red colour). Panels **b, f, l,** and** p** on the middle-left represent DAPI merged with cell-specific markers: CNPase, a myelin marker (**b, c, d**); OLIG2, a marker for oligodendrocytes nuclei (**f, g, h**) 58 k, a Golgi apparatus marker (**l, m, n**); and TPPP, a Golgi outpost marker (**p, q, r**). Panels **c, g, m,** and** q** on the middle-right present merged images from previous columns at the same resolution. Panels **d, h, n,** and** r** on the far right panel provide a higher resolution of the merged picture by 3D reconstruction and in-depth views of signal localization within cellular structures. Protein–protein colocalization of SLC35A2 is evident with CNPase (**c, d**), 58 k (**m, n**), and TPPP (**p, q**) in merged images (yellow signal, white arrowheads). Notably, SLC35A2 colocalizes with TPPP in both perinuclear regions and areas distal to the nucleus (**q, r**). Scale bar in **a**: 10 µm (applies to **b** and **c**); in **d**: 3 µm; in **e**: 10 µm (applies to **f** and **g**); in **h**: 4 µm; in **i**: 4 µm (applies to **l** and **m**); in **n**: 4 µm; in **o**: 2.5 µm (applies to **p** and **q**) and in **r**: 4 µm. Arrowheads in **c, g, m**, and **q** match corresponding regions in the 3D reconstructions (**d, h, n,** and** r**) for spatial reference. Sample IDs: panels **a–d** (CTRL.3, male); Panels **e–h** (CTRL.4, male); Panels **i-n** (CTRL.5, male); Panels **o-r** (CTRL.6, female)

**Fig. 2:**
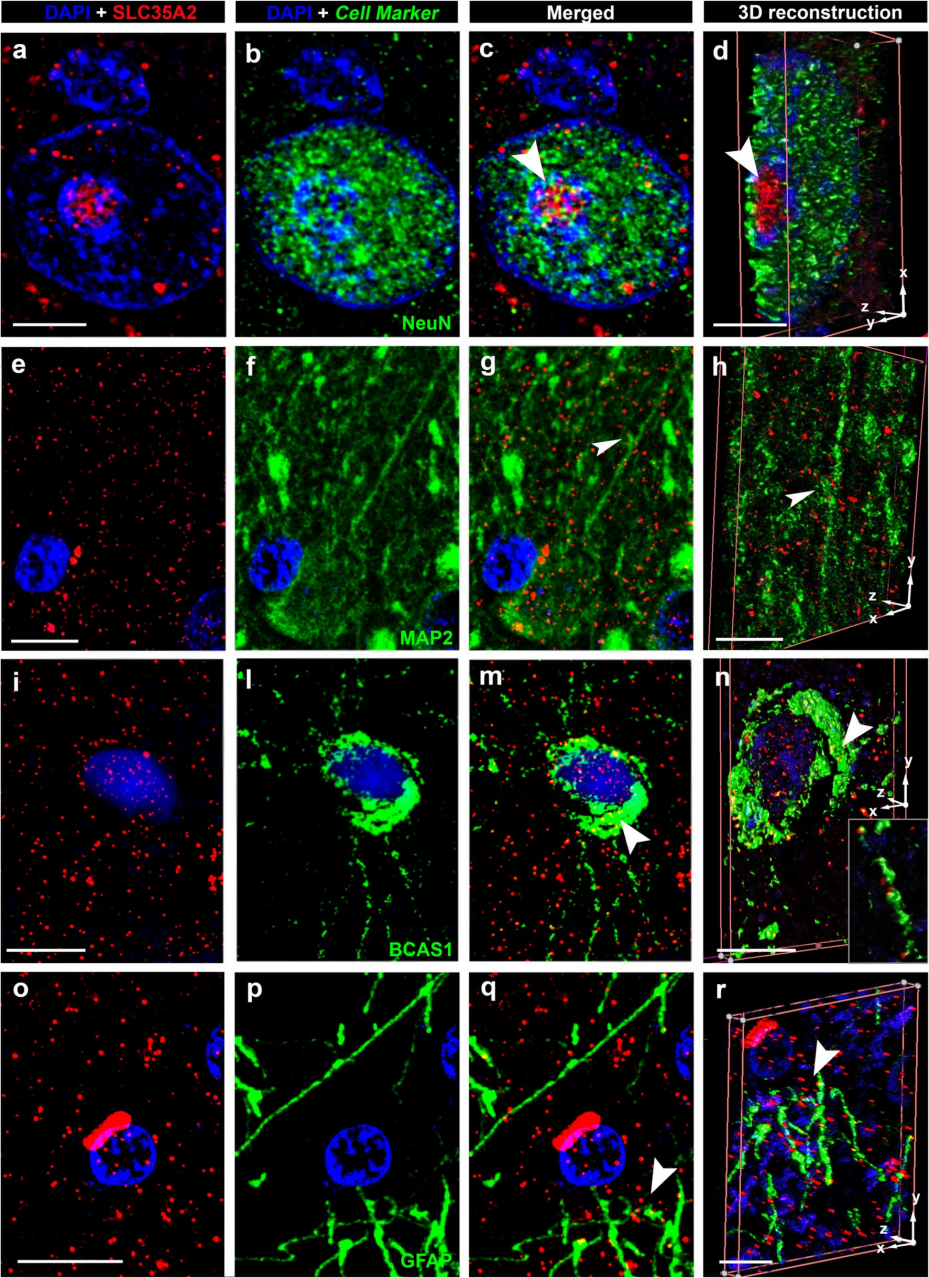
Confocal laser scanning microscopy of SLC35A2 cont’d. Representative Immunofluorescent images are arranged in a 4 × 4-panel format as in Fig. 1. Cell-specific markers include NeuN, a marker for neuronal nuclei (**b, c, d**); MAP2, a microtubule-associated protein in neuronal dendrites (**f, g, h**) BCAS1, a marker for myelinating oligodendrocytes (**l, m, n**); and GFAP, a marker for astrocytes (**p, q, r**). Protein–protein colocalization of SLC35A2 is identified with NeuN (**c, d**) and BCAS1 (**m, n,** see inset in** n**). No clear colocalization is observed with MAP2 (**g, h**) and GFAP (**q, r**). Scale bar in **a**: 5 µm (applies to **b** and **c**), in **d**: 4 µm, in **e**: 10 µm (applies to **f** and **g**), in **h**: 5 µm in **i**: 10 µm (applies to **l** and **m**), in **n**: 12 µm, in **o**: 10 µm (applies to **p** and **q**), and in **r**: 5 µm. Arrowheads in **c, g, m,** and** q** match corresponding regions in the 3D reconstructions (**d, h, n,** and** r**) for spatial reference. Sample IDs: panels **a–d** (CTRL. 3, male); Panels **e–h** (CTRL.4, male) Panels **i–n** (CTRL.23, male); Panels **o-r** (CTRL.5, male)

We applied the newly developed polyclonal antibody to 41 human FFPE brain tissue samples to assess SLC35A2 protein expression and distribution. We performed double labelling with SLC35A2 and major cell markers to examine the protein’s distribution across different cell types and cellular compartments (Figs. 1 and 2). The main protein allocation was observed in perinuclear areas, irrespective of the cell type, and the signal appeared as punctate labelling in a circular pattern. Protein–protein colocalization of SLC35A2 with CNPAse (Fig. 1c, d), 58k (Fig. 1m, n), TPPP (Fig. 1q, r) and BCAS1 (Fig. 2m, n) was observed via confocal microscopy, indicating their co-residence within the same subcellular compartment. No colocalization of SLC35A2 was observed along neuronal processes (as visualized with anti-MAP2 antibodies), nor astroglial processes (GFAP), (Fig. 2)**.**

### 2. Human brain tissue with MOGHE carrying *SLC35A2* nonsense variants reveals protein loss

**Fig. 3:**
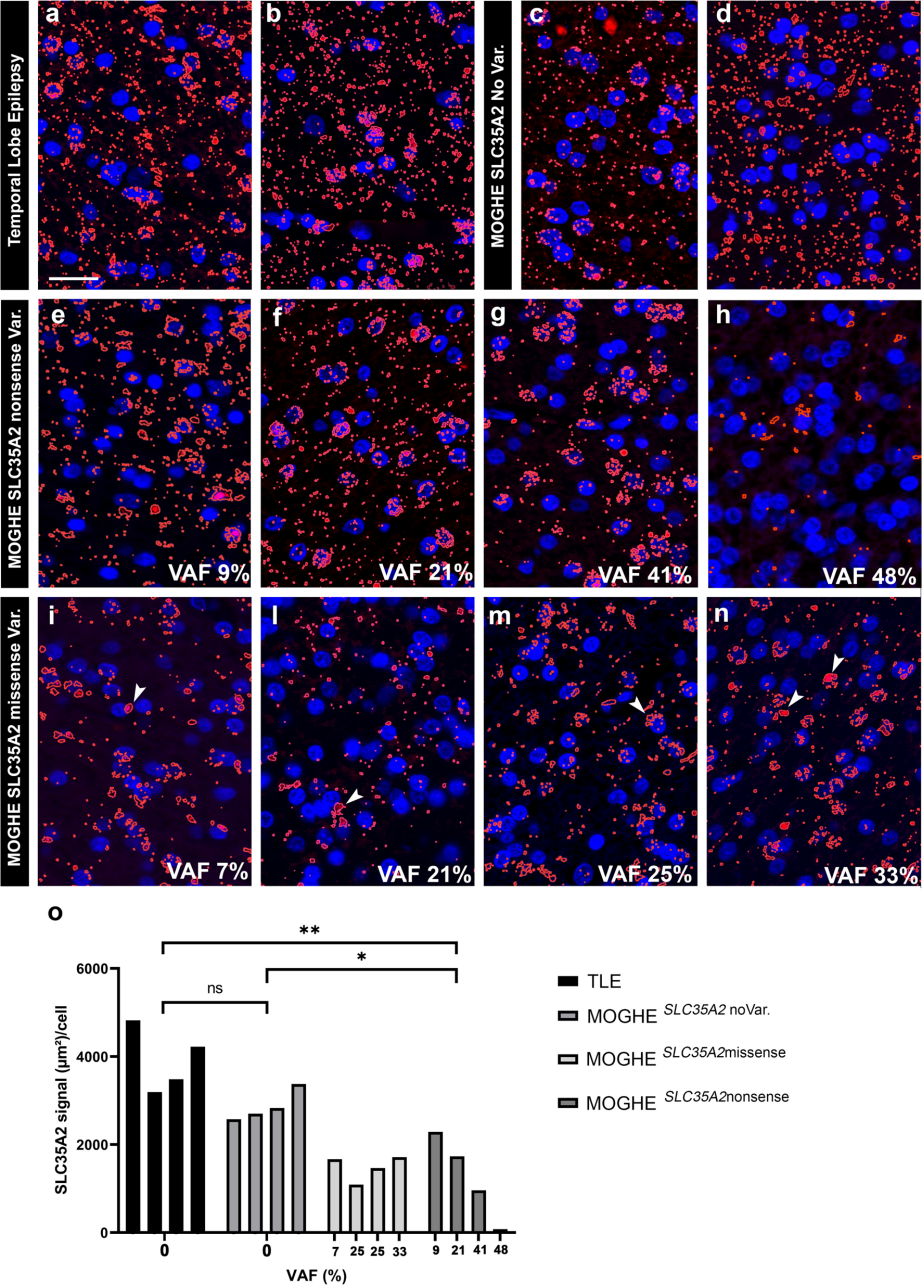
SLC35A2 immunofluorescence staining in MOGHE according to different variant types and their VAF. Immunofluorescence staining of the SLC35A2 epitope obtained from controls and individuals with MOGHE (in red colour). **a, b** TLE control samples showed the characteristic dotted labelling pattern at perinuclear sites as well as scattered throughout the brain tissue (white matter). **c, d:** MOGHE^*SLC35A2noVar.*^ samples shared the same immunolabelling pattern observed in controls. **e–h:** MOGHE^*SLC35A2*nonsense^ samples. Note the progressive loss of the SLC35A2 protein signal when the VAF increased. **i–n:** MOGHE^*SLC35A2*missense^ samples. Note the preferentially perinuclear SLC35A2 immunoreactivity pattern in MOGHE samples with missense variants (white arrowheads in **i, l, m** and** n**). The higher VAF, point not only to a protein loss but also to an apparent altered protein distribution. **o**: SLC35A2 signal quantification in genetically defined groups. The graph displays the ratio of the SLC35A2 protein signal expressed in square µm per cell (*Y* axis) relative to the variant allele frequency of the *SLC35A2* gene (*X* axis). Data are shown for four genetically defined groups: TLE, MOGHE^*SLC35A2*missense^, MOGHE^*SLC35A2*nonsense^, and MOGHE^*SLC35A2*noVar^. Each group included four independent samples. The results revealed a significant loss of SLC35A2 signal per cell in MOGHE^*SLC35A2*nonsense^ compared to the TLE (p 0.0053) and MOGHE^*SLC35A2*noVar.^(p 0.0375). **Scale bar** = 20 µm. **Sample IDs**: Panel **a** (CTRL. 10, female); Panel **b** (CTRL. 5, male); Panel **c** (ID 21, male); Panel **d** (ID 9, male); Panel **e** (ID 50, male); Panel **f** (ID 51, female); Panel **g** (ID 54, male); Panel **h** (ID 47, male); Panel **i** (ID 36, female); Panel **l** (ID 40, male); Panel **m** (ID 38, male); Panel **n** (ID 44, female). All the previous samples plus CTRL.4, male, TLE, CTRL. 6, male, TLE, ID 2, male, MOGHE^*SLC35A2*noVar.^, and ID 8, male, MOGHE^*SLC35A2*noVar^ (IF not shown in the figure) were used to create the statistical analysis in **o**

Single-labelling SLC35A2 staining patterns were compared among control tissues (TLE), MOGHE tissues with no variants, missense variants, and nonsense variants in *SLC35A2*. The results indicated a reduced SLC35A2 protein signal in MOGHE clusters harbouring nonsense mutations compared to both control tissues and those without variants. Differently, MOGHE^*SLC35A2*missense^ tissues did not exhibit a significant loss of protein. However, within these samples, the protein displayed a notable redistribution, with apparent accumulation in perinuclear regions (Fig. 3).

**Fig. 4:**
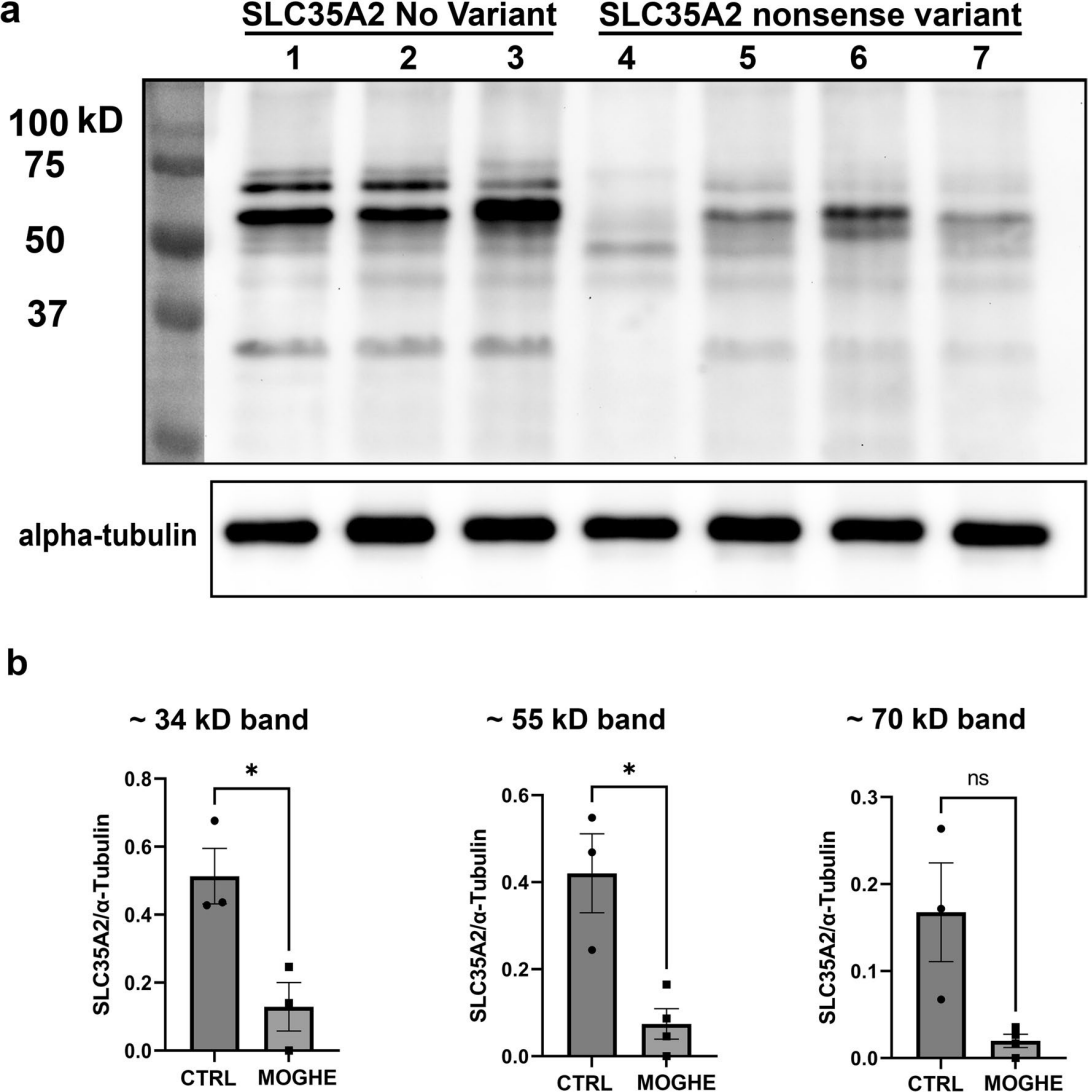
Western blot analysis. **a** Columns 1, 2, and 3 (IDs: CTRL. 1, CTRL.2, CTRL.3, all males) represent individuals with temporal lobe epilepsy (TLE) as controls. Column 4 (ID 47, male), column 5 (ID 54, male), column 6 (ID 52, female), and column 7 (ID 50, male) represent MOGHE^*SLC35A2*nonsense^ samples with a VAF of 48%, 41%, 30%, and 9% respectively. SLC35A2 immunostaining of the blotted protein lysates revealed three distinct bands: the lower band represents the monomeric form of SLC35A2 at approximately 34 kDa, the upper band represents the dimerized form of SLC35A2 at approximately 70 kDa, and the middle band indicates the dimerized form of SLC35A2 with SLC35A3 at approximately 55 kDa. Notably, all MOGHE cases revealed protein loss with the most pronounced reduction in Column 4 (48% VAF). **b** Statistical analysis indicated a significant reduction in protein levels in the lower and middle bands when comparing controls to MOGHE^*SLC35A2*nonsense^. The non-significant difference in the upper band is attributed to high standard deviation among control samples, particularly due to the low detection of the dimerized form in the control sample 3

Western blot analyses revealed specific bands at ~ 34 kD, ~ 55 kD and ~ 70 kD, with the lowest band representing the monomeric form of SLC35A2 and the highest band representing the dimerized form [33, 44]. The middle band of ~ 55 kD likely represent a heterodimer complex formed between SLC35A2 and SLC35A3 [28]. Statistical analysis demonstrated a significant linear correlation between normalized target protein signal intensity and the *SLC35A2* mutation variant allele frequency (VAF). In MOGHE samples with nonsense variants, protein loss was observed, showing a direct correlation with VAF, while samples carrying a missense variant did not exhibit significant protein loss. (Fig. 4).

The results observed in the immunofluorescence (IF) analyses in Fig. 3e, g, and h (Samples ID 50, 54, and 47, respectively) were consistent with those obtained in Western Blot analysis (columns 4, 5, and 7, corresponding to Sample IDs 47, 54, and 50, respectively).

### 3. Increased oligodendroglial cell densities in white matter matches with hypomyelinated areas

Our semi-automated histopathology assessment of Olig2-immunoreactive oligodendroglial cell densities statistically confirmed the recently described manifold increase in all MOGHE cases included in the study (Table 2). However, there was no difference in Olig2-positive cell densities obtained from samples with or without *SLC35A2* variants.

**Table 2:** Oligodendroglial cell density measurements

	Samples (n)	Olig./mm^2^	Min	Max
Autopsy	10	938	763	1141
FCDIIa	10	1450	997	2023
MOGHE^*SLC35A2*noVar^	10	2173	1458	3132
MOGHE^*SLC35A2*missense^	5	2519	1723	3506
MOGHE^*SLC35A2*nonsense^	5	2273	1560	2935

FCDIIa: focal cortical dysplasia type IIa; noVar.: tissue samples without *SLC35A2* variants; Missense: tissue samples positive for *SLC35A2* missense variants; Nonsense: tissue samples positive for *SLC35A2* nonsense variants.; Olig./mm^2^: density of oligodendrocytes nuclei per mm^2^. Min.: values represent the lowest number of oligodendroglial cells detected in a region of interest (ROI) within the group. Max.: values represent the highest number of oligodendroglial cells detected in a region of interest (ROI) within the group

**Fig. 5:**
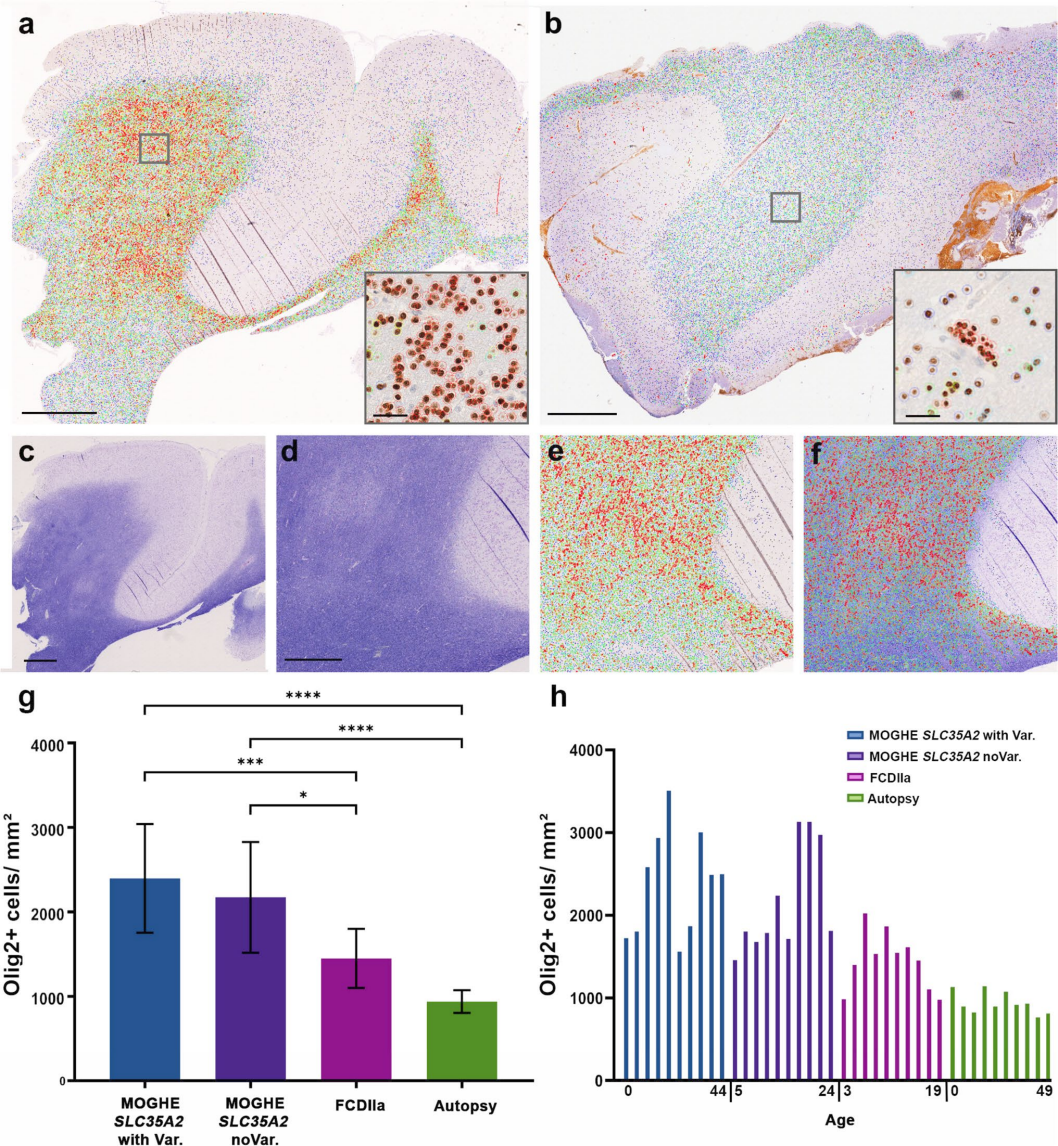
Oligodendroglial cell density heat maps in MOGHE. **a** Heat map showing oligodendrocyte density in a 5-year-old individual with MOGHE^*SLC35A2*noVar.^ (ID 24, female). Regions of high oligodendroglial cell density are depicted in red, with the inset highlighting a cluster. **b** Heat map from an individual with FCDIIa (ID CTRL.14, female, 6 years old), illustrating an even distribution of oligodendrocytes and increased oligodendroglial satellitosis along blood vessels (inset). **c** Nissl-LFB staining from the same MOGHE individual shown in **a** reveals extended patchy discolouration areas, consistent with hypomyelination. **d** Higher magnification of the region shown in **c**, illustrating structural features in more detail. **e** Heat map corresponding to the same area shown in **d**, emphasizing oligodendroglial cell cluster. **f** Overlay of **d** and **e**, confirming alignment between hypomyelination (discolouration in Nissl-LFB) and regions of increased oligodendroglial cell density. **g** Bar graph depicting average oligodendroglial cell density (cells/mm^2^) across four groups: MOGHE with SLC35A2 Variants, MOGHE^*SLC35A2*noVar.^ FCDIIa, and Autopsy. Oligodendrocyte density is significantly higher in MOGHE cases with and without *SLC35A2* variants compared to non-epileptic specimens (p < 0.0001) and FCDIIa (p < 0.001; and p < 0.05). **h** Bar graph representing oligodendroglial cell density (cells/mm^2^) for each of the 40 individual specimens analyzed in panel **g**, categorized into four groups: MOGHE with SLC35A2 Variant (blue), MOGHE^*SLC35A2*noVar.^ (purple), FCDIIa (fuchsia), and Autopsy specimens (green). Digits beneath the *X* axis indicate the lowest and highest ages within each group. **Scale bars**: **a**: 4 mm; **b**: 3 mm; **c**: 2 mm; **d–f**: 1 mm; insets in **a** and b**:** 40 µm

Clusters of oligodendroglial hyperplasia and their preferred localization at the grey-white matter boundary were visualized in density heatmaps (Fig. 5). Densities of Olig2-positive oligodendroglial cells were color-coded from green to red. High-density regions were predominantly located at the grey-white matter boundary in all MOGHE samples, while perivascular oligodendroglial satellitosis was occasionally observed in red in epilepsy controls, such as FCDIIa (insets in Fig. 5b). Overlaying high-density regions with Nissl-LFB stained serial sections from the same FFPE tissue blocks revealed a striking match between red-coded areas and Nissl-LFB discolouration, indicating that clusters of high-density oligodendroglial cells corresponded to areas of hypomyelination. Statistical analyses confirmed a significant increase in Olig2-positive cells in MOGHE individuals, both with and without SLC35A2 variants, compared to FCD IIa and autopsy samples.

**Fig. 6:**
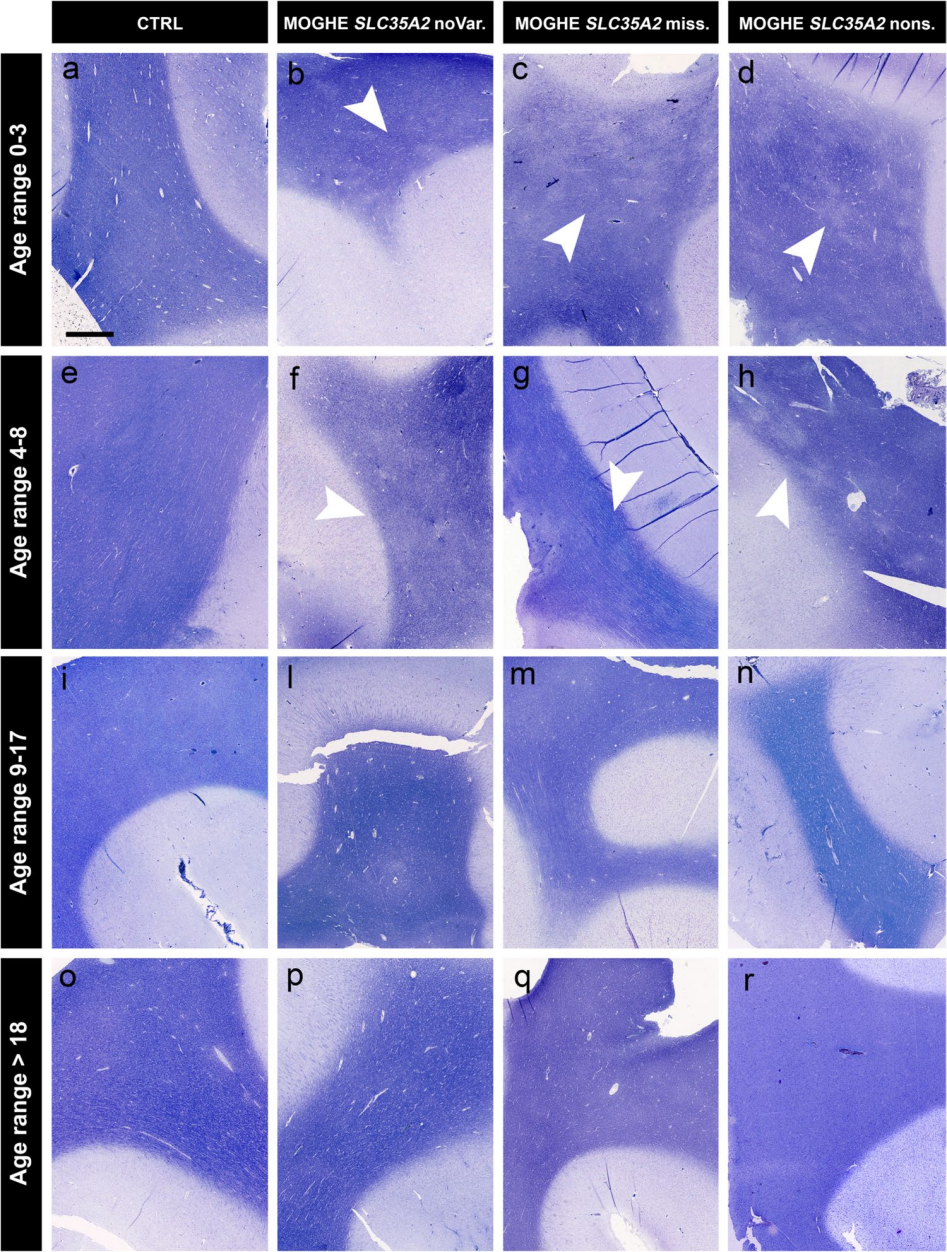
Age-related patchy discolouration of Nissl-LFB in samples with MOGHE. Representative Nissl-Luxol Fast Blue (Nissl-LFB) stained sections showing myelin content across age and genetically defined groups. The figure includes 16 images organized in a 4 × 4 grid, with rows corresponding to four age ranges (0–3 years old: **a–d**, 4–8 years old: **e–h**, 9–17 years old: **i–l**, and over 18 years old: **o–r**) and columns representing different groups (Controls (CTRL): **a, e, i, o**; MOGHE^*SLC35A2*noVar^: **b, f, l, p**; MOGHE^*SLC35A2*missense^: **c, g, m, q**; MOGHE^*SLC35A2*nonsense^: **d, h, n, r**). Nissl-LFB staining revealed patchy areas of low myelin content in MOGHE patients aged 0–8 years (**b, c, d, f, g, h**). These patchy discolorations were observed in MOGHE patients and were absent in the control group, which included autopsy samples and tissue from individuals with FCDIIa. The hypomyelination pattern became progressively less pronounced with age in MOGHE patients (**l, m, n, p, q, r**). Sample IDs: Panel **a** (CTRl.23); Panel **b** (ID 30, male); Panel **c** (ID 39, male); Panel **d** (ID 51, female); Panel **e** (CTRL. 14); Panel **f** (ID 31, male); Panel **g** (ID 42, male); Panel **h** (ID 57, male); Panel **i** (CTRL. 25); Panel **l** (ID 2, male), Panel **m** (ID 36, female), Panel **n** (ID 45, male); Panel **o** (CTRL. 28), Panel **p** (ID 22, male), Panel **q** (ID 32, male), Panel **r** (ID 59, female)

Nissl-LFB staining revealed a patchy pattern of hypomyelination in tissue samples from patients with MOGHE, which was particularly pronounced in younger age groups. These features were most prominent in patients aged 0–3 years old and remained detectable up to the age of 8 years. This pattern became less apparent in patients aged 9–17 years old and was hardly detectable in individuals over 18 years of age (Fig. 6).

### 4. Ultrastructural analyses confirm hypomyelination in MOGHE

**Fig. 7:**
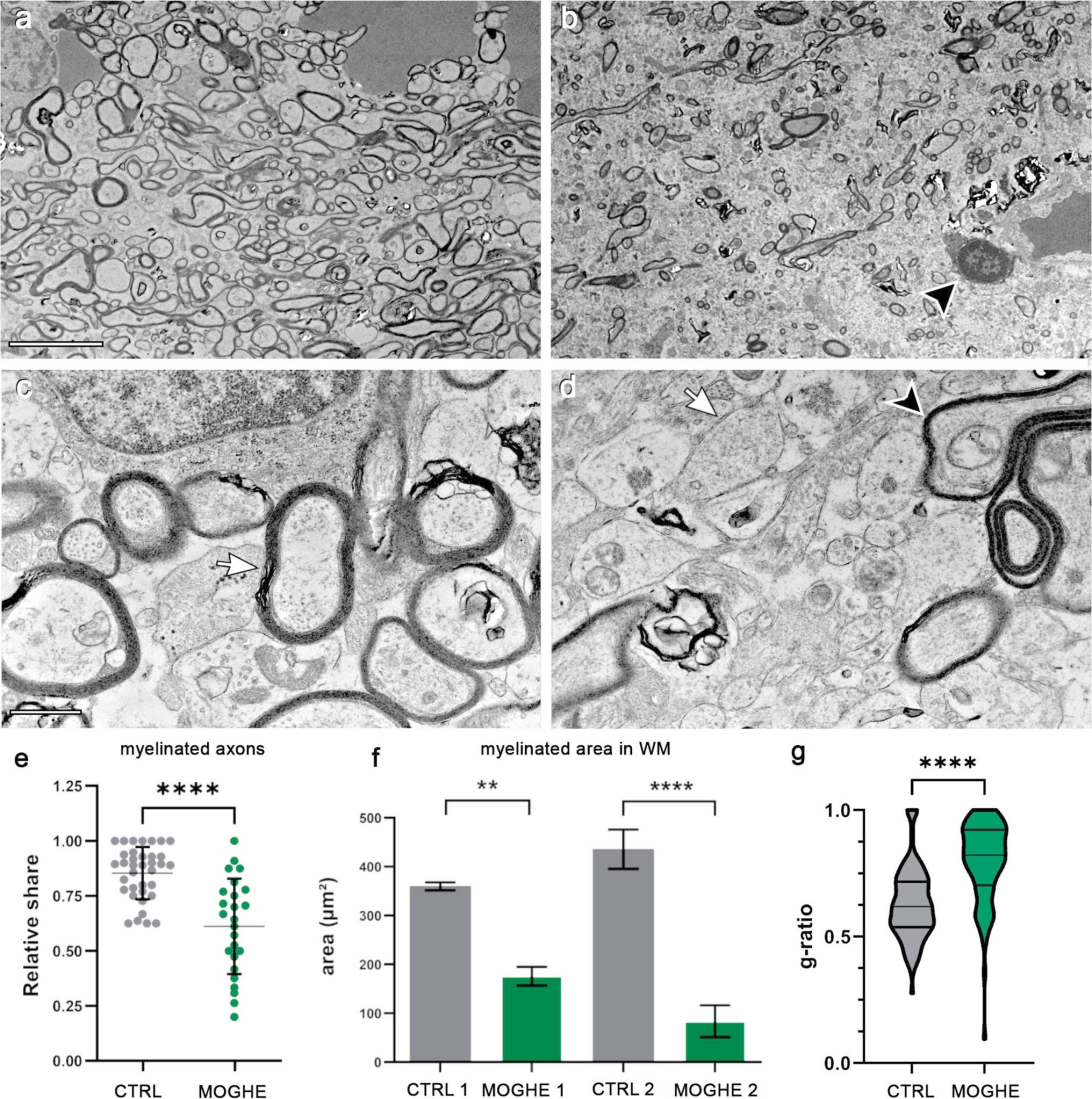
Electron micrographs of surgically resected MOGHE. **a** The perilesional area to MOGHE revealed high-density axonal cross sections at the level of the white matter (ID 7, male, 5 years old, MOGHE^*SLC35A2noVar.*^). **b** A MOGHE white matter lesion area with reduced density of myelinated axons and hypomyelinated axonal cross sections (ID 43, female, 44 years old, MOGHE^*SLC35A2*missense^ with 8% VAF). **c** Higher magnification of MOGHE perilesional white matter area. The arrow indicates an axon with a tightly packed, multi-layered myelin sheath (ID 43, as in **b**). **d** Higher magnification of axonal cross-sections from MOGHE lesion area (ID 7, as, in **a**) showing several single-layered, hypomyelinated axons (white arrow) and an axon with a loosened, or outfolding myelin sheath (black arrowhead) [14, 39]. **e** Samples representing perilesional areas to MOGHE (CTRL) with normal oligodendroglial cell densities, showed an average myelination of 80% (gray). MOGHE lesion areas (MOGHE) exhibited a significant decrease, with a relative share of myelinated axons of 60% (green). Each dot represents an EM frame (magnification fold: 1500x; frame area: 1mm^2^). **f** Histogram plots depict the absolute area of myelin per µm^2^ in MOGHE lesions (green) compared to controls (grey), measured from the digital micrograph. **g** G-ratio in lesional MOGHE areas showed a significant difference (p < 0.0001) when studying 100 axons from four different samples compared to their perilesional areas. The median g-ratio in lesional MOGHE areas was 0.822 compared to 0.619 in perilesional areas indicating hypomyelination. Scale bars in **a-b**: 5µm; Scale bars in **c-d** 1µm

Histological and IHC evidence of hypomyelination in MOGHE was then corroborated at the ultrastructural level. Clusters of oligodendroglial hyperplasia were identified on araldit-embedded semithin sections and observed at 1500 × –10,000 × magnification. Digital micrographs were then compared with those taken from adjacent areas showing normal oligodendroglial cell densities, as identified on the same semithin sections. MOGHE areas revealed a predominance of axons with single-layered myelin (Fig. 7b, d), contrary to the higher prevalence of multi-layered axons within the perilesional zones (Fig. 7a, c). Interestingly, hypomyelinated areas also showed the presence of oligodendrocytes with heterochromatin-enriched nuclei (Fig. 7b**,** black arrowhead). We semi-quantitatively measured the relative share of myelinated axons within the MOGHE and perilesional areas from the same ultrathin sections, as described elsewhere [35], to assess the magnitude of hypomyelination. All MOGHE samples revealed a significant reduction in myelinated axons compared to the perilesional area (Fig. 7e). In addition, our semi-quantitative measurement of the myelin area (µm^2^) covering the selected areas of interest was also significantly different with up to a fourfold decrease within MOGHE lesion areas compared to the control (Fig. 7f). Due to the low number of samples we were unable to assess differences between samples with no *SLC35A2* variant, nonsense variants, and missense variants.

### 5. Age-related reduction of BCAS1-immunoreactive oligodendrocytes differs in MOGHE individuals and controls

**Fig. 8:**
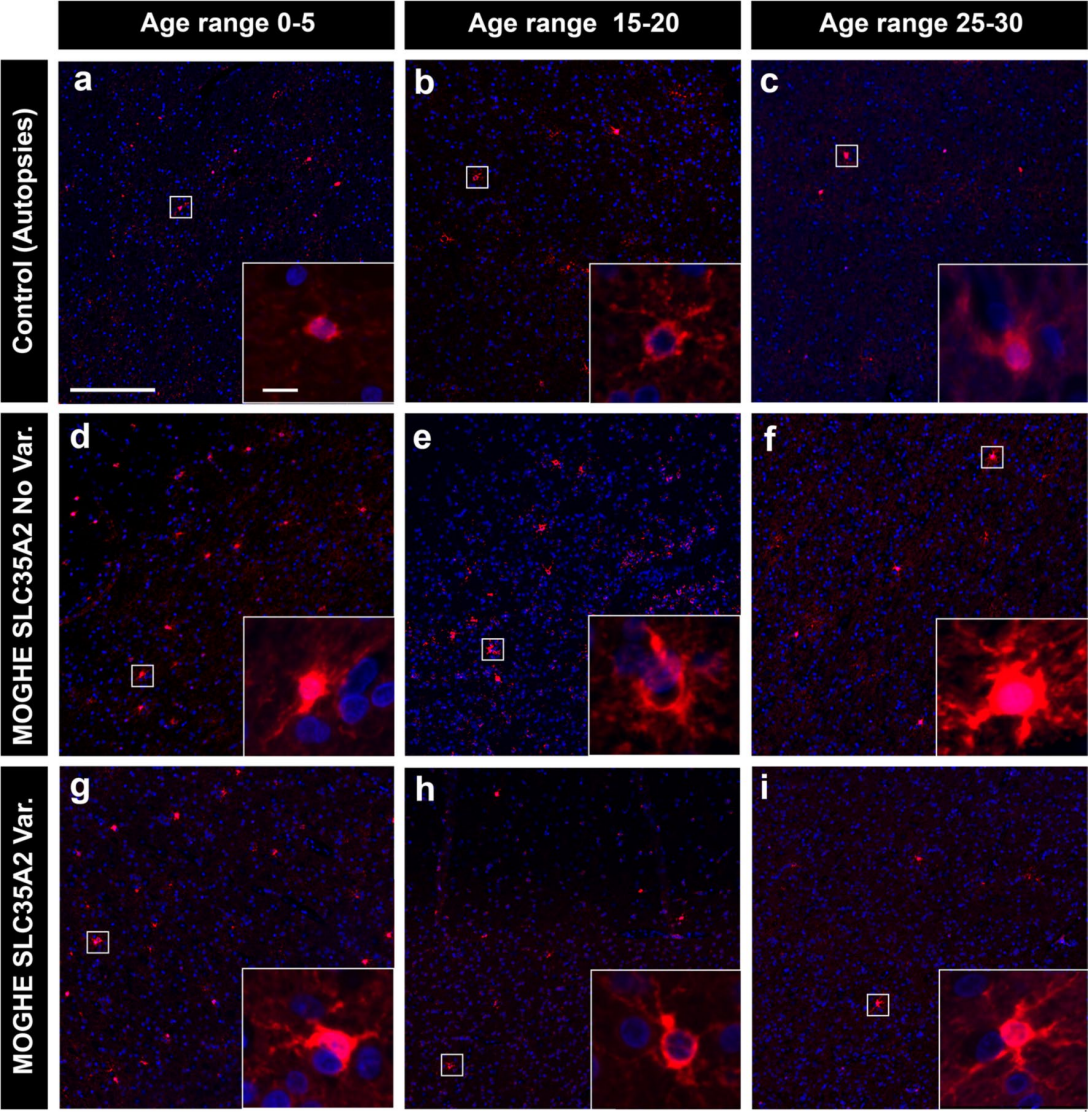
BCAS1—immunoreactivity in MOGHE compared to controls. Representative images of brain samples stained with BCAS1, illustrating differences between controls (top row, **a–c**), MOGHE^*SLC35A2*noVar.^ (middle row, **d–f**), and MOGHE with variants (bottom row, **g–i**). Columns correspond to distinct age groups: 0–5 years old (first column), 15–20 years old (second column), and 25–30 years old (third column). The images revealed a significant increase in BCAS1-positive cell expression in MOGHE samples compared to controls, the latter having a consistently low BCAS1-immunolabelling. An age-dependent decrease in BCAS1-positive cells was observed in both MOGHE groups. Insets provided higher magnification views of BCAS1-positive cells. Scale bar in **a**: 200 μm applies to all main images and in inset, **a**: 10 μm, applies to all insets. **Sample IDs**: Panel **a** (CTRL. 23, male); Panel **b** (CTRL. 27, male); Panel **c** (CTRL. 28, male); Panel **d** (ID 31, male); Panel **e** (ID 2, male); Panel **f** (ID 22, male); Panel **g** (ID 54, male); Panel **h** (ID 36, female); Panel **i** (ID 32, male)

**Fig. 9:**
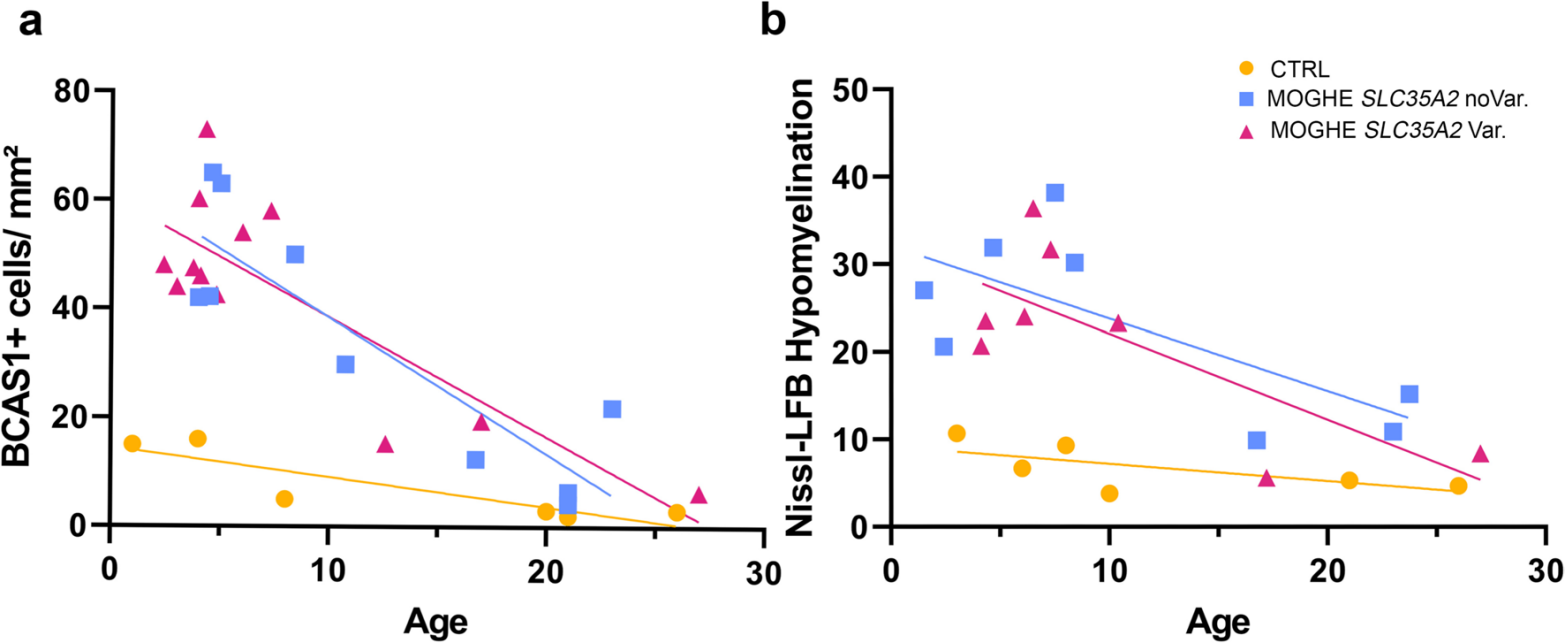
Age-related reduction of BCAS1-immunoreactive oligodendrocytes. **a** Age-related reduction of BCAS1-immunoreactive oligodendrocytes. Yellow circles: Controls including autopsies and individuals with TLE (*n* = 6); Blue square: Individuals with MOGHE^*SLC35A2*noVar.^ (*n* = 10); Pink Triangles: Individuals with MOGHE with missense and nonsense *SLC35A2* variants (*n* = 12); Symbol legend applies for both panel **a** and **b**. There is a significant inverse correlation between the patient's age at epilepsy surgery and the number of BCAS1-immunoreactive cells per 1 mm^2^ of white matter. Pearson correlation: CTRL (*r* = − 0.8709; 95% confidence interval = − 0.9857 to − 0.2024; *R* squared = 0.7585; *P* (two-tailed) = 0,0009); MOGHE^*SLC35A2*noVar.^ (*r* = − 0.874; 95% confidence interval = − 0.9699 to − 0.5435; *R* squared = 0.7639; *P* (two-tailed) = 0,0239); MOGHE^*SLC35A2*Var.^ (*r* = − 0.8211; 95% confidence interval = − 0.9482 to − 0.4676; *R* squared = 0.6743; *P* (two-tailed) = 0.0011). **b** Age-related reduction of patchy areas of hypomyelination as indicated by Nissl-LFb staining (see also Fig. 6). Controls (*n* = 6); Individuals with MOGHE^*SLC35A2*noVar.^ (*n* = 8); Individuals with MOGHE with missense and nonsense *SLC35A2* variants (*n* = 8). Nissl-LFB Hypomyelination (*Y* axis) indicates the relative intensity difference of each sample in Nissl-LFB staining related to the patient's age at surgery in years (*X* axis) in MOGHE groups and TLE individuals, and age of death for the autopsy samples included in the control group. Pearson correlation: CTRL (*r* = − 0.6564; 95% confidence interval = − 0.9578 to 0.3321; *R* squared = 0.4309, *P* (two-tailed) = 0.1568); MOGHE^*SLC35A2*noVar.^ (*r* = − 0.7106; 95% confidence interval = − 0.9430 to − 0.01181; *R* squared = 0.5049; *P* (two-tailed) = 0.0482); MOGHE^*SLC35A2*Var^ (*r* = − 0.7457; 95% confidence interval = − 0.9508 to − 0.08652; *R* squared = 0.5561; *P* (two-tailed) = 0.0337)

Next, we aimed to better understand the age-related MRI signal changes in patients with MOGHE as previously described [20, 29]. Using antibodies against BCAS1 (Brain Enriched myelin-associated protein 1, previously known as Breast Carcinoma Amplified Sequence 1), a marker for oligodendrocytes active in myelin formation [15, 22, 41] we conducted a semi-quantitative analysis of BCAS1 immunoreactivity. In this intermediary developmental stage, oligodendrocytes undergo a transitional phase from precursor cells to mature oligodendrocytes, while actively participating in myelin production. Our results revealed significantly higher BCAS1-positive cell densities in MOGHE patients compared to age-matched non-epileptic controls (Figs. 8, 9). The mean density of BCAS1-positive cells in MOGHE areas without *SLC35A2* variants was 37.5 cells per mm^2^ (mean age at surgery: 8.99 years; *n* = 10), compared to 41.5 cells per mm^2^ in MOGHE samples with *SLC35A2* variants (mean age at surgery: 8.27 years; *n* = 12), with no significant difference between these groups. In contrast, the non-epilepsy control group showed a lower mean density of 10.2 BCAS1-positive cells per mm^2^ in the white matter (mean age: 10.2 years; *n* = 6).

Our analysis indicated an age-dependent decrease in the density of BCAS1-positive oligodendrocytes in MOGHE, with younger patients exhibiting significantly higher densities than older individuals (Fig. 9a). Additionally, we observed an age-dependent reduction in hypomyelination in the MOGHE group. Hypomyelination was assessed by comparing staining intensities within MOGHE lesion and perilesional areas on the same sections, showing notable changes in younger individuals, that reduced with age (Fig. 9b). Still, no significant differences were found between the genetically defined MOGHE groups.

### 6. Excess of heterotopic neurons is a common feature in MOGHE

**Fig. 10:**
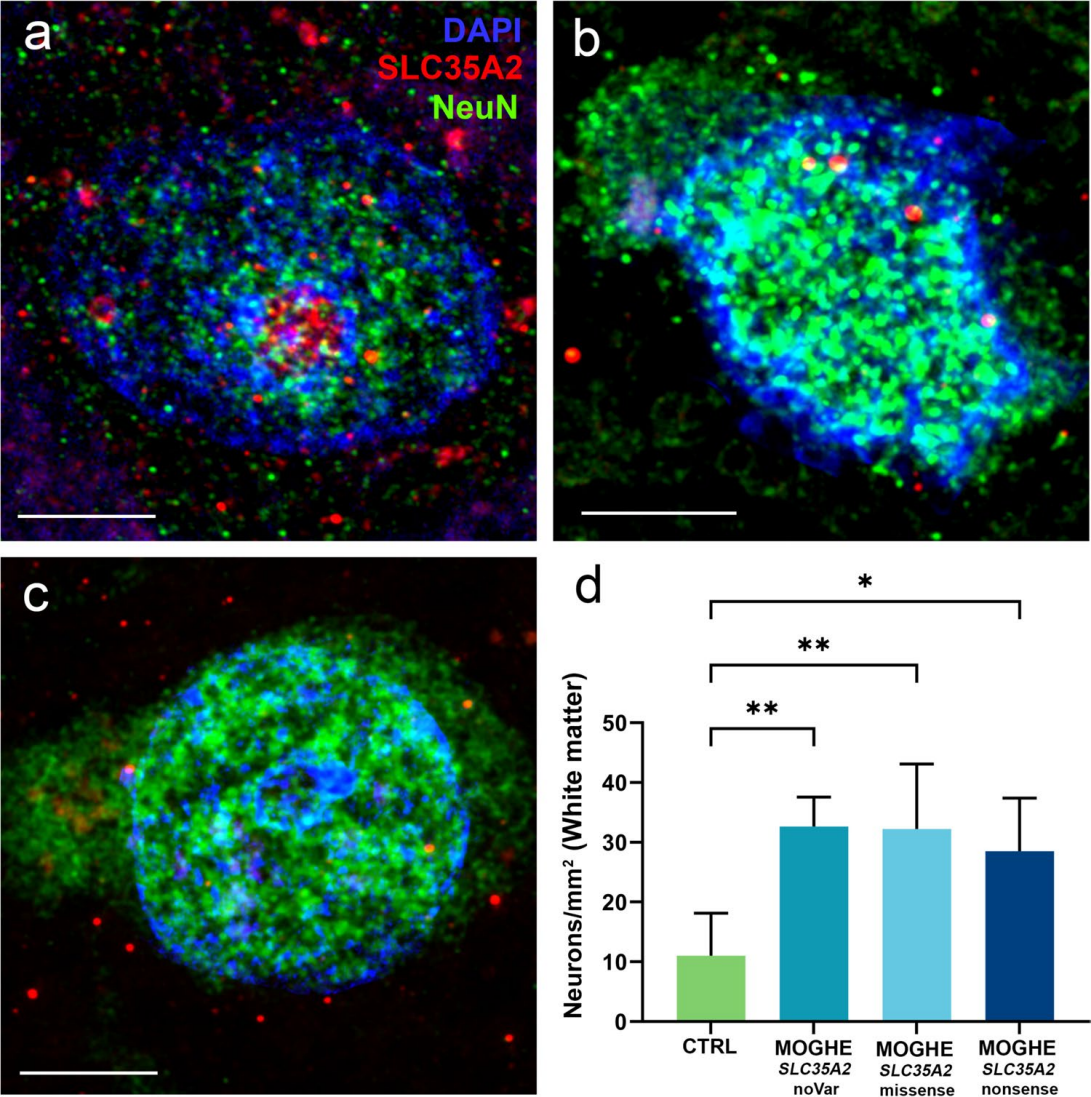
SLC35A2 expression in cortical vs. heterotopic neurons in MOGHE. **a** Cortical neuron from a control case (ID CTRL. 9). **b** heterotopic neuron from the white matter of a MOGHE^*SLC35A2*noVar^. (ID 29). **c** heterotopic neuron from the white matter of a MOGHE^*SLC35A2*missense^ (ID 40). **d** The figure displays the density of neurons within the white matter (measured per mm^2^) across different sample groups (*n* = 4 for each group). In control cases, the average neuronal count is approximately 12 neurons per mm^2^. In contrast, MOGHE cases showed a significantly elevated density, with neuronal counts increasing up to threefold compared to controls. Scalebars—**a**: 5 µm **b**: 5 µm **c**:7 µm

We investigated the presence of heterotopic neurons in the white matter of MOGHE^*SLC35A2*noVar.^, MOGHE^*SLC35A2*nonsense^, and MOGHE^*SLC35A2*missense^ samples. Our results did not show a significant difference in the content of heterotopic neurons in the white matter among the genetically defined MOGHE groups. However, every MOGHE group showed a significant increase of heterotopic neurons within the white matter, compared to controls (Fig. 10).

## Discussion

Our results are consistent with the notion that (1) in the human brain, the SLC35A2 protein is localized within the Golgi apparatus. Furthermore, the signal detected away from cell nuclei closely matches markers for Golgi outposts, predominantly along oligodendroglial cell processes; (2) a significant SLC35A2 protein loss was observed in brain tissue samples from individuals with MOGHE carrying nonsense variants. Protein loss was also observed in tissue samples with missense variants, but did not reach statistical significance; (3) hypomyelination was confirmed at the ultrastructural level in regions with increased oligodendroglial cell densities; (4) hypomyelination patterns were reduced in older individuals with MOGHE, as indicated by Nissl-LFB staining. In parallel, there was a reduction in BCAS1-immunoreactive oligodendrocytes in older individuals compared to the increased number of BCAS1-immunoreactive oligodendrocytes at early ages. High-density oligodendroglial cell clusters and hypomyelination were observed across all MOGHE individuals, irrespective of the presence of *SLC35A2* variants.

We first examined the cellular localization of the SLC35A2 protein in the neocortex and white matter of the human brain, allowing us to observe the protein in the context of tissue architecture (Figs. 1, 2). As expected, SLC35A2 was associated with the Golgi apparatus; however, we also observed its presence along the oligodendroglial cell processes, indicated by the colocalization of SLC35A2 with TPPP, a marker for Golgi outposts [16] and CNPase. Noteworthy, the punctate staining was decreased in MOGHE^*SLC35A2*nonsense^ compared to MOGHE^*SLC35A2*noVar.^ or control samples. We semi-quantitatively measured the SLC35A2 signal across four groups of individuals: TLE, MOGHE^*SLC35A2*noVar.^, MOGHE^*SLC35A2*missense^, and MOGHE^*SLC35A2*nonsense^. In samples carrying missense variants, protein levels appeared largely preserved, although, the protein predominantly accumulated near the nucleus i.e., likely detained within the Endoplasmic Reticulum and Golgi apparatus [23]. In contrast, nonsense variants resulted in a pronounced loss of protein, with SLC35A2 levels showing an inverse correlation with variant allele frequency (VAF), underlying a variant-dependent loss-of-function effect.

One point of discussion when analyzing these variants is the impact of nonsense variants on protein expression and the influence of sex differences, as these factors can interact and conceal the interpretation. Nonsense variants affect protein expression depending on their position within the coding sequence. Variants near the C-terminus typically result in truncated proteins detectable by antibodies directed against epitopes from the N-terminus, whereas variants near the N-terminus often trigger nonsense-mediated decay (NMD), leading to transcript degradation and loss of full-length protein [40]. Mid-gene variants may result in the production of alternative isoforms of the protein [11]. Due to the mosaic nature of the variant within tissue with MOGHE and the varying variant allele frequencies, it is challenging to predict whether the affected proteins undergo NMD or are simply truncated, as we also detected protein from non-mutated cells. Nonetheless, our antibody is able to detect several forms and isoforms supporting our major conclusion of SLC35A2 protein loss in MOGHE tissue with pathogenic *SLC35A2* variants. Given the X-linked nature of SLC35A2, sex differences also play a role. In males with a single X chromosome, the variant allele typically results in the expression of the altered protein. In females, mosaic X-inactivation can lead to variable expression depending on the frequency of the variant allele on the active X chromosome. These sex-specific differences in VAF may influence protein expression and disease manifestation, and will require further attention.

The ideal approach to validate the specificity of the self-designed antibody involves testing with knockout cell lines or tissues. However, such knockout cell lines were not readily accessible and the development of knockout (KO) or conditional knockout (cKO) *SLC35A2* models presents significant challenges [13, 47]. Nevertheless, the antibody applied to Western blotting detected proteins with approximate molecular weights of 34 kDa, 55 kDa, and 70 kDa. The bands corresponding to the lowest and highest molecular weights represented the monomeric and dimeric forms of SLC35A2, respectively [33, 44]. The intermediate band at 55 kDa was indicative of the dimerized form of SLC35A2 in complex with SLC35A3 [28]. Notably, the Western blot data (Fig. 4) revealed a loss of SLC35A2 protein in surgical MOGHE samples, which contained a mixture of affected and unaffected cells. These samples included cells with properly functioning SLC35A2, with variant allele frequencies (VAF) ranging from 9 to 48%. These findings provide the first evidence of partial SLC35A2 protein loss in human MOGHE brain tissue and support the specificity of the antibody in detecting SLC35A2 isoforms. However, we acknowledge the limitation of the self-designed antibody in distinguishing between variant and non-variant forms of the SLC35A2 protein, which may restrict its ability to detect specific isoforms or post-translational modifications.

We have previously observed areas of hypomyelination co-registering with the clusters of oligodendroglial hyperplasia, in particular at the grey-white matter boundary (Fig. 5; [36]). These findings likely reflect the commonly described laminar MRI signal intensity changes at the grey-white matter boundary of young individuals with histopathologically confirmed MOGHE [20, 29]. In our current cohort, we compared the MOGHE groups for the staining intensity of myelinated fibres in the white matter. These were significantly decreased in all MOGHE cases but there was no difference between genetically defined groups. To substantiate the hypomyelination phenotype of MOGHE we collected tissue samples also for ultrastructural analysis in three patients. The results confirmed the hypomyelination and revealed the presence of thin myelin sheaths at different ages. This observation may point to delayed early myelination in individuals with MOGHE at different ages [38] (Fig. 7). The density of BCAS1-positive oligodendrocytes was significantly higher in young MOGHE individuals compared to older individuals from our cohort (Figs. 8, 9a). BCAS1 antibodies were used to characterize a distinct subset of generative and regenerative, BCAS1-positive oligodendrocytes when they undergo a transitional phase from precursor cells to mature oligodendrocytes, while actively participating in myelin production [15, 41]. The precise function of the BCAS1 protein remains to be elucidated but it is hypothesized to govern intracellular transport mechanisms in oligodendrocytes by interaction with the dynein light chain [32]. The absence of BCAS1 in knockout mouse models induced a schizophrenia-like behaviour [13] suggesting an important role for normal myelination and brain function [21]. These findings well represent the structural correlate of the MRI changes at the grey-white matter boundary. Yet, none of these studies revealed significant differences between genetically defined subgroups.

The occurrence of similar histopathology findings for oligodendroglial hyperplasia, hypomyelination and an age-related attenuation in MOGHE individuals with and without *SLC35A2* mosaicism suggested the existence and convergence of other pathogenic variants and molecular pathways. These questions are not only of academic interest as individuals may become resistant to currently available anti-seizure medications or do not benefit from surgical resections. Knowledge of the disease-underlying genetic pathway offers the opportunity for targeted treatment as recently shown in a pilot trial using d-galactose supplementation in patients with MOGHE [1]. Oral administration of d-galactose has been shown to result in clinical improvement not only for SLC35A2-CDG patients [45] but also for individuals with *SLC35A2* brain mosaicism, such as in MOGHE [1]. The variation in protein expression in relation to VAF and variant type described herein may influence the efficacy of such targeted therapies. Specifically, the degree of protein loss and the VAF could modulate the response to D-galactose supplementation suggesting the potential value of targeted treatment strategies tailored to the genetic profile in each individuum.

In conclusion, MOGHE is defined histopathologically and neuroradiologically by oligodendroglial hyperplasia and an age-related attenuation of hypomyelination at the grey-white matter junction of the neocortex. In half of individuals, we detected variable brain mosaicism for the *SLC35A2* gene and protein loss in individuals carrying nonsense variants. However, we observed a similar degree of oligodendroglial hyperplasia and hypomyelination in MOGHE individuals independent of the *SLC35A2* variant status. These results suggested other pathomechanisms to be involved. To open future avenues for targeted treatment, cooperative research studies will be necessary to address epileptogenic lesions of the white matter and their underlying molecular pathways.

## Supplementary Information

Below is the link to the electronic supplementary material.

Supplementary file1 (DOCX 2906 KB)
