# Supplementary material for: Human brain tissue with MOGHE carrying somatic SLC35A2 variants reveal aberrant protein expression and protein loss in the white matter
Source: Acta Neuropathol. 2025 Mar 5;149(1):23. doi: 10.1007/s00401-025-02858-7 (PMC11882685; doi:10.1007/s00401-025-02858-7)
Supplement: Supplementary file 1 — Supplementary file1 (DOCX 2906 KB) [file 401_2025_2858_MOESM1_ESM.docx]

**Supplementary Material**

**Supplementary Table 1: Case Cohorts**

**A. MOGHE Cohort**

| **ID** | **Sex** | **Surgery** | **Onset** | **Region** | **SLC35A2 Variant** | **p.code** | **Variant type** | **VAF** | **Outcome (m)** | **Previous Report** |
| --- | --- | --- | --- | --- | --- | --- | --- | --- | --- | --- |
| 1. | M | 10,75 | 1,91 | h.-L | No Var. |  |  | - | 1a (34m) | Bosselmann et al. 2024[8] |
| 2. | M | 16,75 | 1,41 | F-R | No Var. |  |  | - | 1a (46m) | Bosselmann et al. 2024[8] |
| 3. | M | 5 | 1,5 | F-R | No Var. |  |  | - | 1a (12m) | Leu et al. 2023[25] |
| 4. | F | 23,08 | n.a. | T-L | No Var. |  |  | - | 1a | Leu et al. 2023[25] |
| 5. | M | 23 | n.a. | F-R | No Var. |  |  | - | n.a. | Leu et al. 2023[25] |
| 6. | M | 3,16 | 0,16 | F-R | No Var. |  |  | - | 1a (12m) | Leu et al. 2023[25] |
| 7. | M | 5 | n.a. | h.-R | No Var. |  |  | - | n.a. | Leu et al. 2023[25] |
| 8. | M | 7,58 | 1,16 | F-R | No Var. |  |  | - | n.a. | Leu et al. 2023[25] |
| 9. | M | 35,5 | n.a. | T-R | No Var. |  |  | - | 3a | Leu et al. 2023[25] |
| 10. | M | 5,58 | 3 | F-L | No Var. |  |  | - | n.a. | Leu et al. 2023[25] |
| 11. | F | 8,5 | 6,33 | F-L | No Var. |  |  | - | 2c (43) | Leu et al. 2023[25] |
| 12. | M | 7,16 | 0,58 | F-R | No Var. |  |  | - | n.a. | Leu et al. 2023[25] |
| 13. | F | 11,16 | 3 | hem.-R | No Var. |  |  | - | n.a. | Leu et al. 2023[25] |
| 14. | F | 4,83 | 3 | F-L | No Var. |  |  | - | 1a (18m) | Lopez-Rivera et al. 2023[27] |

| **ID** | **Sex** | **Surgery** | **Onset** | **Region** | **SLC35A2 Variant** | **p.code** | **Variant type** | **VAF** | **Outcome (m)** | **Previous Report** |
| --- | --- | --- | --- | --- | --- | --- | --- | --- | --- | --- |
| 15. | F | 21,83 | 10 | T | No Var. |  |  | - | 3a | Lopez-Rivera et al. 2023[27] |
| 16. | M | 5,5 | 0,75 | F-L | No Var. |  |  | - | 1a (60m) | Lopez-Rivera et al. 2023[27] |
| 17. | F | 4,5 | 0,16 | F-R | No Var. |  |  | - | 1a | Lopez-Rivera et al. 2023[27] |
| 18. | F | 10,58 | 0,33 | F-L | No Var. |  |  | - | 4a (78m) | Leu et al. 2023[25] |
| 19. | F | 7,33 | 1,5 | F-L | No Var. |  |  | - | 1a (17m) | Leu et al. 2023[25] |
| 20. | M | 3 | n.a. | hem.-L | No Var. |  |  | - | n.a. | Leu et al. 2023[25] |
| 21. | M | 4 | 0,33 | F-R | No Var. |  |  | - | 1a (28m) | Lopez-Rivera et al. 2023[27] |
| 22. | M | 21,25 | 3,5 | F-R | No Var. |  |  | - | 1a | Lopez-Rivera et al. 2023[27] |
| 23. | F | 18,75 | 0,75 | F-R | No Var. |  |  | - | 3a (215m) | Lopez-Rivera et al. 2023[27] |
| 24. | F | 21,58 | 12 | F-R | No Var. |  |  | - | n.a. | Lopez-Rivera et al. 2023[27] |
| 25. | F | 9,16 | n.a. | F-R | No Var. |  |  | - | n.a. | Leu et al. 2023[25] |
| 26. | M | 4,5 | 2,25 | F-L | No Var. |  |  | - | 2(12m) | Lopez-Rivera et al. 2023[27] |
| 27. | M | 7,75 | 0,58 | F-R | No Var. |  |  | - | 4b (23m) | Lopez-Rivera et al. 2023[27] |
| 28. | M | 23,91 | 2 | F-L | No Var. |  |  | - | 1a (76m) | Leu et al. 2023[25] |
| 29. | M | 5,33 | 0,41 | F-R | No Var. |  |  | - | n.a. | Bosselmann et al. 2024[8] |
| 30. | M | 3,33 | 0,5 | F-R | No Var. |  |  | - | 1a (86m) | Leu et al. 2023[25] |
| 31. | M | 4,41 | 0,58 | TPO-L | No Var. |  |  | - | 4a (106m) | Lopez-Rivera et al. 2023[27] |

| **ID** | **Sex** | **Surgery** | **Onset** | **Region** | **SLC35A2 Variant** | **p.code** | **Variant type** | **VAF** | **Outcome (m)** | **Previous Report** |
| --- | --- | --- | --- | --- | --- | --- | --- | --- | --- | --- |
| 32. | M | 27 | 3 | TO-L | c.665_667del | p.K222del | missense | n.a | 3a | Lopez-Rivera et al. 2023[27] |
| 33. | F | 7,12 | 0,5 | F-R | c.935C>T | p.S312F | missense | 8,00% | n.a. | Lopez-Rivera et al. 2023[27] |
| 34. | F | 59 | 24 | F-R | c.515T>C | p.L172P | missense | 3,00% | 2a(12m) | Lopez-Rivera et al. 2023[27] |
| 35. | M | 2,41 | 0,16 | F-L | c.533G>T | p.G178V | missense | 29,40% | 1a(18m) | Lopez-Rivera et al. 2023[27] |
| 36. | F | 17,41 | 0,58 | F-R | c.905C>T | p.S302F | missense | 7,00% | 4a(29m) | Lopez-Rivera et al. 2023[27] |
| 37. | M | 22 | 12 | F-R | c.844G>A | p.G282R | missense | 9,00% | n.a. | Leu et al. 2023[25] |
| 38. | M | 4 | 0,5 | F-L | c.322C>T | p.Q108X | missense | 25,00% | 1a | Barba et al. 2023[3] |
| 39. | M | 2,58 | 0,83 | F-R | c.206C>T | p.T69L | missense | 14,00% | 1a(76m) | Barba et al. 2023[3] |
| 40. | M | 9,58 | 1,5 | T-L | c.223G>A | p.E75K | missense | 21,30% | 1a(36m) | Leu et al. 2023[25] |
| 41. | F | 3,5 | 0,58 | F-L | c.640G>C | p.G214R | missense | n.a | 1a(85m) | Barba et al. 2023[3] |
| 42. | M | 4 | n.a | F-L | c.844G>A | p.G282R | missense | 25,50% | n.a. | Leu et al. 2023[25] |
| 43. | F | 44 | 15 | TPO-L | c.640G>C | p.G214R | missense | 8,00% | 3a(24m) | Lopez-Rivera et al. 2023[27] |
| 44. | F | 3,5 | 0,58 | F-R | c.935C>T | p.S312F | missense | 33,00% | 1a(19m) | Lopez-Rivera et al. 2023[27] |
| 45. | M | 12,75 | 7 | F-R | c.335_339dupCGCTC | p.K114Rfs*32 | nonsense | 52,00% | 1b(33m) | Barba et al. 2023[3] |
| 46. | M | 3 | 1,91 | T-L | c.547C>T | p.Q183X | nonsense | 8,40% | 1a(31m) | Leu et al. 2023[25] |
| 47. | M | 6,5 | 2 | T-L | c.832C>T | p.Q278* | nonsense | 48,40% | 1a(26m) | Lopez-Rivera et al. 2023[27] |
| 48. | F | 6,5 | 3 | F-L | c.322C>T | p.Q108X | nonsense | 8,00% | 4b (24m) | Lopez-Rivera et al. 2023[27] |
| **ID** | **Sex** | **Surgery** | **Onset** | **Region** | **SLC35A2 Variant** | **p.code** | **Variant type** | **VAF** | **Outcome (m)** | **Previous Report** |
| 49. | M | 4 | 0,58 | h.-L, FT-L | c.921_947delinsT | p.S308Wfs*106 | nonsense | 10,60% | 3a (24m) | Lopez-Rivera et al. 2023[27] |
| 50. | M | 6,16 | 1,58 | h.-R,F-R | c.580_616dupCCACTGGATCAGAACCCTGGGGCAGGCCTGGCAGCCG | p.V206Afs*28 | nonsense | 9,00% | 1a(28m) | Lopez-Rivera et al. 2023[27] |
| 51. | F | 3,75 | 0,41 | h.-L | c.603_606dupAGGC | p.L203Rfs*20 | nonsense | 21,00% | 1a(51m) | Barba et al. 2023[3] |
| 52. | F | 4,25 | 0,33 | F-L | c.359_360delTC | p.L120Hfs*7 | nonsense | 30,00% | 4a(25m) | Lopez-Rivera et al. 2023[27] |
| 53. | M | 7,33 | 3 | F-R, FTPO | c.675dupA | p.G226Rfs*29 | nonsense | 40,00% | 1a(48m) | Lopez-Rivera et al. 2023[27] |
| 54. | M | 4,83 | 1,5 | F-R | c.113delT | p.I38fs | nonsense | 41,00% | 1a(25m) | Lopez-Rivera et al. 2023[27] |
| 55. | M | 4,41 | 0,33 | F-R | c.569_572delGAGG | p.Gly190Alafs*158 | nonsense | 41,00% | 3a(36m) | Barba et al. 2023[3] |
| 56. | M | 4,41 | 0,5 | FT-R | c.634_635del | p.S212Lfs*9 | nonsense | 8,40% | 1a(91m) | Leu et al. 2023[25] |
| 57. | M | 5,75 | 0,58 | F-L | c.634_635del | p.S212Lfs*9 | nonsense | 22,90% | 1b(14m) | Leu et al. 2023[25] |
| 58. | M | 5,41 | 0,5 | FTPO-R | c.424C>T | p.Q142X | nonsense | 19,40% | 1a(53m) | Leu et al. 2023[25] |
| 59. | F | 43,5 | 12 | F-R | c.364_365insC,  c.366delinsTCTC | p.Y122Sfs*6,  p.Y122_T123insL | nonsense | n.a | 3a(12m) | Lopez-Rivera et al. 2023[27] |

**Legend to supplementary Table 1A**: h.: hemisphere; R: right; L: left; F: Frontal; P: parietal; T: Temporal, O: occipital; VAF: Variant Allele Frequency. ^1^Refers to age at surgery, ^2^Refers to age of epilepsy onset,^3^ Epileptogenic Brain region, ^4^  SLC35A2 Variant type ^5^ protein code ^6^ Variant Allele Frequency,  ^7^ Outcome after surgery according to Engel surgical outcome scale, Engel class I-IV, in months (m).

**B. Control Cohort**

| **ID** | **Sex** | **Age** | **Diagnosis** | ***SLC35A2* Var.** | **Previous Report** |
| --- | --- | --- | --- | --- | --- |
| CTRL. 1 | M | 50 | TLE | n.a. | n.a. |
| CTRL. 2 | M | 36 | TLE | n.a. | n.a. |
| CTRL. 3 | M | 20 | TLE | n.a. | n.a. |
| CTRL. 4 | M | 12 | TLE | No Var. | n.a. |
| CTRL. 5 | M | 28 | TLE | n.a. | n.a. |
| CTRL. 6 | F | 18 | TLE | n.a. | n.a. |
| CTRL. 7 | M | 12 | TLE | n.a. | n.a. |
| CTRL. 8 | M | 20 | TLE | No Var. | n.a. |
| CTRL.9 | M | 5 | TLE | n.a. | n.a. |
| CTRL. 10 | F | 14 | TLE | n.a. | n.a. |
| CTRL. 11 | M | 3 | FCD IIa | No Var. | Bosselmannet al. [8] |
| CTRL. 12 | F | 3 | FCD IIa | No Var. | López-Rivera et al. [27] |
| CTRL. 13 | M | 4 | FCD IIa | No Var. | Niestroj et al. [31] |
| CTRL. 14 | F | 6 | FCD IIa | n.a. | n.a. |
| CTRL. 15 | F | 9 | FCD IIa | n.a. | n.a. |
| CTRL. 16 | M | 11 | FCD IIa | No Var. | López-Riveraet al. [27] |
| CTRL. 17 | M | 12 | FCD IIa | No Var. | López-Rivera et al. [27] |
| CTRL. 18 | M | 18 | FCD IIa | No Var. | López-Rivera et al. [27] |
| CTRL. 19 | F | 18 | FCD IIa | No Var. | López-Rivera et al. [27] |
| CTRL. 20 | F | 19 | FCD IIa | negQC. | n.a. |
| CTRL. 21 | F | 0 | Autopsy | n.a. | n.a. |
| CTRL. 22 | M | 0,58 | Autopsy | n.a. | n.a. |
| CTRL. 23 | M | 3,58 | Autopsy | n.a. | n.a. |
| CTRL. 24 | M | 5 | Autopsy | n.a. | n.a. |
| CTRL. 25 | M | 17 | Autopsy | n.a. | n.a. |
| CTRL. 26 | M | 20 | Autopsy | n.a. | n.a. |
| CTRL. 27 | M | 22 | Autopsy | n.a. | n.a. |
| CTRL. 28 | M | 26 | Autopsy | n.a. | n.a. |
| CTRL. 29 | F | 26 | Autopsy | n.a. | n.a. |
| CTRL. 30 | M | 49 | Autopsy | n.a. | n.a. |

**Legend to supplementary Table 1B**: **CTRL**: Control, **M**: male, **F**: female, **TLE**: Temporal Lobe Epilepsy, **FCDIIa**: Focal Cortical Dysplasia type IIa, **SLC35A2 Var**.: mutational status for SLC35A2, **n.a**.: non-available. **negQC.**: negative quality control; low DNA quality did not allow further analysis.

| **Primary Antibody** | **Clone** | **Host** | **Company** | **Dilution** |
| --- | --- | --- | --- | --- |
| SLC35A2 | Polyclonal | Rabbit | Abcam (ab222854) | 1:50 |
| SLC35A2 | Polyclonal | Rabbit | PSL GmbH* | 1:50 |
| CNPase | Monoclonal | Mouse | Millipore (MAB326) | 1:200 |
| NeuN | Monoclonal | Mouse | Millipore (MAB377) | 1:1500 |
| MAP2 | Monoclonal | Mouse | UNIL** | 1:100 |
| GFAP | Monoclonal | Mouse | Dako | 1:500 |
| Olig2 | Monoclonal | Mouse | Millipore | 1:100 |
| NaBC1 (BCAS1) | Monoclonal | Mouse | Santa Cruz Biotechnology | 1:150 |
| 58K | Monoclonal | Mouse | Abcam | 1:500 |
| TPPP | Polyclonal | Goat | Invitrogen | 1:150 |
| **Secondary Antibody** | **Clone** | **Host** | **Company** | **Dilution** |
| ALEXA Fluor 555 Goat-anti-rabbit | Polyclonal | Goat | Invitrogen | 1:100 |
| ALEXA Fluor 647 Goat-anti-rabbit | Polyclonal | Goat | Invitrogen | 1:100 |
| ALEXA Fluor 555 Goat-anti-mouse | Polyclonal | Goat | Invitrogen | 1:100 |
| ALEXA Fluor 647 Goat-anti-mouse | Polyclonal | Goat | Invitrogen | 1:100 |
| ALEXA Fluor 555 Donkey-anti-goat | Polyclonal | Donkey | Invitrogen | 1:100 |

**Supplementary Table 2: Primary and secondary antibodies used in immunofluorescence analyses**

* Peptide Specialty Laboratories GmbH, Germany. Peptide Specialty Laboratories GmbH is a subsidiary of Reaction Biology Europe

** Produced by Universitè de Lausanne, Facultè de Biologie et de Mèdecine, Départment de Biologie Cellulaire et de Morphologie (DBCM)

**Supplementary Table 3: Control and MOGHE samples used in each experiment and relative statistical analyses**

| **Experiments** | | | | | | **Statistical analyses** | | | |
| --- | --- | --- | --- | --- | --- | --- | --- | --- | --- |
| **IF** | **WB** | **Olig. Cell** | **Nissl-LFB** | **EM** | **IF - Bcas1** | **Fig. 3** | **Fig. 5** | **Fig. 9** | **Fig. 10** |
| 1. | CTRL. 1 | 1. | 2. | 7. | 1. | 2. | 1. | 1. | 9. |
| 2. | CTRL. 2 | 11. | 22. | 29. | 2. | 8. | 11. | 2. | 29. |
| 7. | CTRL. 3 | 14. | 30. | 39. | 11. | 9. | 14. | 11. | 40. |
| 8. | 27. | 16. | 31. |  | 14. | 21. | 16. | 14. |  |
| 9. | 33. | 17. | 32. |  | 16. | 36. | 17. | 16. |  |
| 11. | 34. | 22. | 36. |  | 17. | 38. | 22. | 17. |  |
| 14. | 36. | 24. | 39. |  | 22. | 40. | 24. | 22. |  |
| 16. | 38. | 27. | 42. |  | 26. | 44. | 27. | 26. |  |
| 17. | 42. | 28. | 45. |  | 27. | 47. | 28. | 27. |  |
| 19. | 43. | 29. | 51. |  | 28. | 50. | 29. | 28. |  |
| 21. | 44. | 32. | 57. |  | 31. | 51. | 32. | 31. |  |
| 22. | 47. | 33. | 59. |  | 32. | 54. | 33. | 32. |  |
| 24. | 48. | 36. | CTRL. 14 |  | 35. | CTRL. 4 | 36. | 35. |  |
| 25. | 49. | 39. | CTRL. 23 |  | 36. | CTRL. 5 | 39. | 36. |  |
| 26. | 50. | 43. | CTRL. 25 |  | 38. | CTRL. 6 | 43. | 38. |  |
| 27. | 52. | 47. | CTRL. 28 |  | 44. | CTRL. 10 | 47. | 44. |  |
| 28. | 54. | 50. |  |  | 45. |  | 50. | 45. |  |
| 29. |  | 51. |  |  | 47. |  | 51. | 47. |  |
| 31. |  | 53. |  |  | 50. |  | 53. | 50. |  |
| 32. |  | 59. |  |  | 51. |  | 59. | 51. |  |
| 33. |  | CTRL. 11 |  |  | 52. |  | CTRL. 11 | 52. |  |
| 35. |  | CTRL. 12 |  |  | 54. |  | CTRL. 12 | 54. |  |
| 36. |  | CTRL. 13 |  |  | CTRL. 1 |  | CTRL. 13 | CTRL. 1 |  |
| 38. |  | CTRL. 14 |  |  | CTRL. 4 |  | CTRL. 14 | CTRL. 4 |  |
| 39. |  | CTRL. 15 |  |  | CTRL. 22 |  | CTRL. 15 | CTRL. 22 |  |
| 40. |  | CTRL. 16 |  |  | CTRL. 23 |  | CTRL. 16 | CTRL. 23 |  |
| 41. |  | CTRL. 17 |  |  | CTRL. 27 |  | CTRL. 17 | CTRL. 27 |  |
| 42. |  | CTRL. 18 |  |  | CTRL. 28 |  | CTRL. 18 | CTRL. 28 |  |
| 43. |  | CTRL. 19 |  |  |  |  | CTRL. 19 |  |  |
| 44. |  | CTRL. 20 |  |  |  |  | CTRL. 20 |  |  |
| 45. |  | CTRL. 21 |  |  |  |  | CTRL. 21 |  |  |
| 46. |  | CTRL. 22 |  |  |  |  | CTRL. 22 |  |  |
| 47. |  | CTRL. 23 |  |  |  |  | CTRL. 23 |  |  |
| 50. |  | CTRL. 24 |  |  |  |  | CTRL. 24 |  |  |
| 51. |  | CTRL. 25 |  |  |  |  | CTRL. 25 |  |  |
| 52. |  | CTRL. 26 |  |  |  |  | CTRL. 26 |  |  |
| 53. |  | CTRL. 27 |  |  |  |  | CTRL. 27 |  |  |
| 54. |  | CTRL. 28 |  |  |  |  | CTRL. 28 |  |  |
| 55. |  | CTRL. 29 |  |  |  |  | CTRL. 29 |  |  |
| 57. |  | CTRL. 30 |  |  |  |  | CTRL. 30 |  |  |
| 59. |  |  |  |  |  |  |  |  |  |
| CTRL. 1 |  |  |  |  |  |  |  |  |  |
| CTRL. 2 |  |  |  |  |  |  |  |  |  |
| CTRL. 3 |  |  |  |  |  |  |  |  |  |
| CTRL. 4 |  |  |  |  |  |  |  |  |  |
| CTRL. 5 |  |  |  |  |  |  |  |  |  |
| CTRL. 6 |  |  |  |  |  |  |  |  |  |
| CTRL. 7 |  |  |  |  |  |  |  |  |  |
| CTRL. 8 |  |  |  |  |  |  |  |  |  |
| CTRL.9 |  |  |  |  |  |  |  |  |  |
| CTRL. 10 |  |  |  |  |  |  |  |  |  |

**Legend to supplementary Table 3**: **IF**: samples used for immunofluorescence stainings; **WB:** samples used for Western Blotting analyses, **Olig. Cell**: cases stained for OLIG2 and examined for oligodendroglial cell density in the white matter and used for the generation of Heatmaps. **Nissl-LFB:** cases stained with Nissl-Luxol Fast Blue for evaluation of the myelin content. **EM**: available samples for ultrastructural analyses via Electron Microscopy, **IF- Bcas1**: samples used for immunofluorescence stainings with BCAS1, **Fig 3, 5, 8 and 9**: samples used for the generation of the statistical analyses for each Figure.

**Supplementary Fig. 1: Comparison of SLC35A2 signal between commercially available and newly designed antibodies**


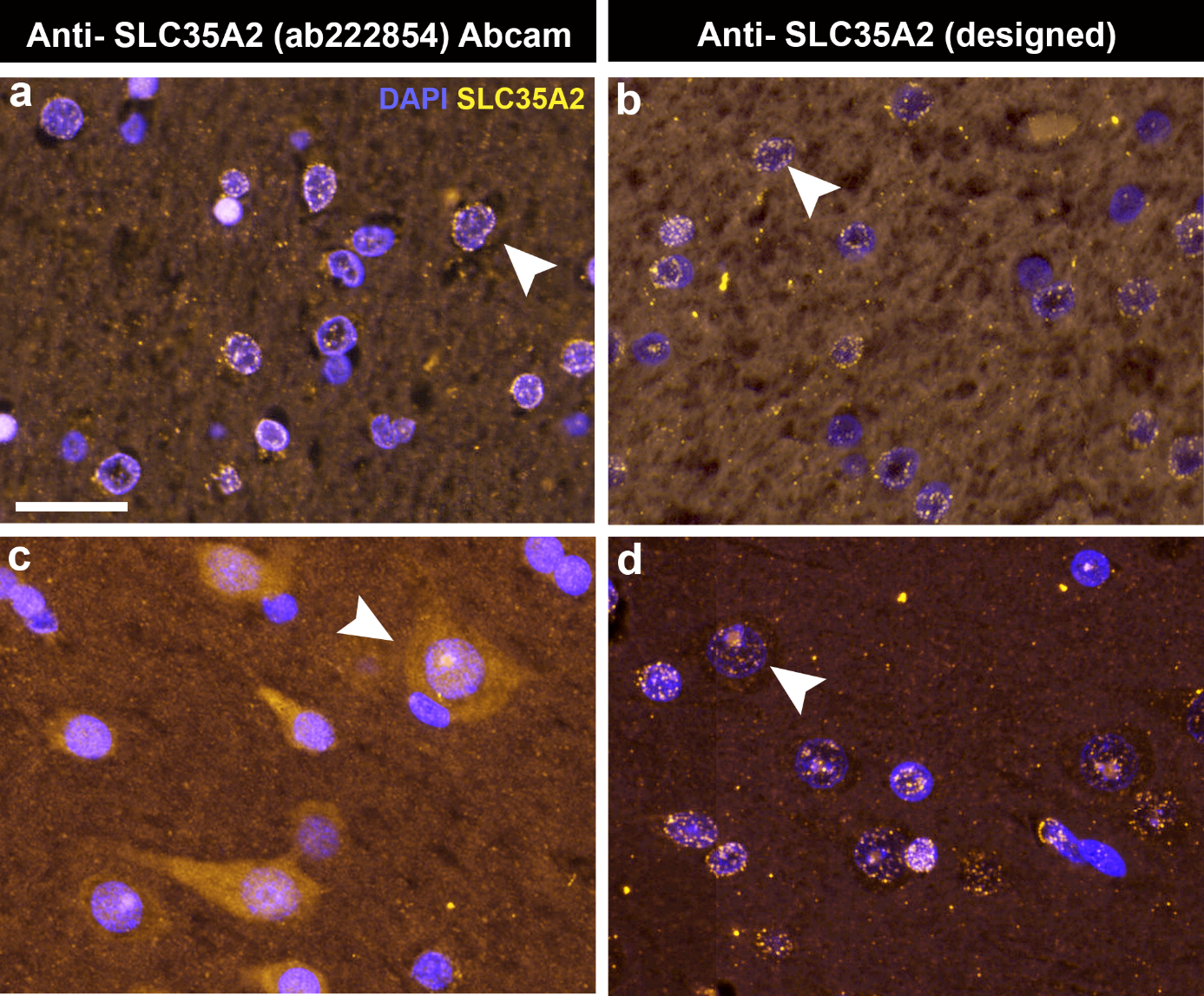

**Legend to supplementary Figure 1**. Comparison of SLC35A2 immunoreactivity between the newly designed on the right panel and commercially available antibodies on the left panel. All stainings were performed on non-MOGHE (control) brain tissue samples from a 14-year-old individual with Temporal Lobe Epilepsy. In the white matter (**a, b**), both antibodies show a comparable and dotted SLC35A2 immunoreactivity pattern (arrows) aligned to cell nuclei of likely glial cell lineage. In the neocortex (**c, d**), the signal obtained with the anti-SLC35A2 antibody (ab222854) revealed a shallow staining of neuronal cell bodies (**c**) compared to a predominantly dotted immunoreactivity pattern in both neuronal (arrow in **d**) and glial nuclei. We interpret the staining pattern of our new antibody, therefore, as similar or more specific than the commercial one. Scale bar: 25 μm.
